# Mutations in the Cholesterol Transporter Gene *ABCA5* Are Associated with Excessive Hair Overgrowth

**DOI:** 10.1371/journal.pgen.1004333

**Published:** 2014-05-15

**Authors:** Gina M. DeStefano, Mazen Kurban, Kwame Anyane-Yeboa, Claudia Dall'Armi, Gilbert Di Paolo, Heather Feenstra, Nanette Silverberg, Luis Rohena, Larissa D. López-Cepeda, Vaidehi Jobanputra, Katherine A. Fantauzzo, Maija Kiuru, Marija Tadin-Strapps, Antonio Sobrino, Anna Vitebsky, Dorothy Warburton, Brynn Levy, Julio C. Salas-Alanis, Angela M. Christiano

**Affiliations:** 1 Department of Genetics and Development, Columbia University, New York, New York, United States of America; 2 Department of Dermatology, Columbia University, New York, New York, United States of America; 3 Department of Pediatrics, Columbia University Medical Center, New York, New York, United States of America; 4 Department of Pathology and Cell Biology, Columbia University, New York, New York, United States of America; 5 Taub Institute for Research on Alzheimer's Disease and the Aging Brain, Columbia University Medical Center, New York, New York, United States of America; 6 St. Luke's-Roosevelt Hospital Center, New York, New York, United States of America; 7 Centro Dermatológico Pascua, Mexico City, Mexico; 8 New York Presbyterian Hospital, New York, New York, United States of America; 9 Basic Science, Universidad de Monterrey, Nueva Leon, Mexico; Max-Delbrück-Center for Molecular Medicine, Germany

## Abstract

Inherited hypertrichoses are rare syndromes characterized by excessive hair growth that does not result from androgen stimulation, and are often associated with additional congenital abnormalities. In this study, we investigated the genetic defect in a case of autosomal recessive congenital generalized hypertrichosis terminalis (CGHT) (OMIM135400) using whole-exome sequencing. We identified a single base pair substitution in the 5′ donor splice site of intron 32 in the ABC lipid transporter gene *ABCA5* that leads to aberrant splicing of the transcript and a decrease in protein levels throughout patient hair follicles. The homozygous recessive disruption of *ABCA5* leads to reduced lysosome function, which results in an accumulation of autophagosomes, autophagosomal cargos as well as increased endolysosomal cholesterol in CGHT keratinocytes. In an unrelated sporadic case of CGHT, we identified a 1.3 Mb cryptic deletion of chr17q24.2-q24.3 encompassing *ABCA5* and found that *ABCA5* levels are dramatically reduced throughout patient hair follicles. Collectively, our findings support *ABCA5* as a gene underlying the CGHT phenotype and suggest a novel, previously unrecognized role for this gene in regulating hair growth.

## Author Summary

Inherited hypertrichoses represent a group of hair overgrowth syndromes that are extremely rare in humans and have remained an area of great interest to evolutionary geneticists since they are considered to be recurrences of an ancestral phenotype. These syndromes often present with additional congenital abnormalities including bone, heart and dental defects; thus, it is crucial to identify the mechanisms and genes underlying the pathology. Copy number variants (CNVs) have previously been reported in several cases of congenital generalized hypertrichosis terminalis (CGHT) with a minimal overlapping region of 555 kb encompassing four genes. However, no point mutations in these or any other single genes have been described to underlie the CGHT phenotype. In this study, we report the first loss-of-function mutation in an ABC transporter, *ABCA5* and identified an additional copy number variant in a separate case that lies within the minimal common region. We found high levels of *ABCA5* expression in both epithelial and mesenchymal compartments of human and mouse hair follicles, and in CGHT patients, this expression is significantly reduced or completely lost. ABCA5 is a lysosomal protein, and its loss-of-function compromises the integrity of lysosomes and leads to an intra-endolysosomal accumulation of cholesterol. Importantly, our findings support a novel role for *ABCA5* in regulating hair growth.

## Introduction

Inherited hypertrichosis, first described in the sixteenth century, is characterized by hair growth that is excessive for the body site and age of an individual and is independent of androgen stimulation [1], [2]. These syndromes are categorized as ectodermal dysplasias and are often associated with additional congenital defects, including cardiomyopathy, gingival hyperplasia, and craniofacial malformations [3]. The genetic basis of these syndromes remained largely elusive until recently, when our group and others reported several chromosomal rearrangements, copy number variants (CNVs) and position effects involving genes associated with autosomal dominant, recessive, sporadic, and X-linked forms of hypertrichosis [4]–[10].

We previously reported a position effect on the zinc finger transcription factor *TRPS1* in Ambras syndrome hypertrichosis in humans and the *Koala* hypertrichosis phenotype in mice, where *Trps1* expression was decreased at the sites of pathology for the phenotype [5]. More recently, we and others elucidated the genetic basis of X-linked hypertrichosis [9],[10], resulting from large interchromosomal insertions on the X chromosome. We found that a position effect occurs on a distant gene, *FGF13* ***,*** whose expression was markedly and selectively reduced in patient hair follicles, suggesting a novel role for this growth factor in hair follicle growth and cycling [10].

In the autosomal dominant form of CGHT, we identified a series of duplications of chromosome 17q24.2-q24.3 and reported a position effect on the *SOX9* gene, situated ∼1 Mb downstream of these variants, and found *SOX9* expression was markedly reduced throughout the follicular epithelium of patient hair follicles [4]. In a separate report of CNVs in the same region of chromosome 17q24.2-q24.3, four CNVs were identified in several cases of CGHT [6], with an overlapping minimal region of 555 kb encompassing four genes: *ABCA6, ABCA10, ABCA5, and MAP2K6,* suggesting that disruption of one of these genes may contribute to the CGHT phenotype. Despite the identification of CNVs and/or position effects in this region of chromosome 17q24.2-q24.3, no point mutations in these or any other single genes have been described to underlie the CGHT phenotype.

In this study, we investigated the genetic basis of autosomal recessive CGHT (OMIM135400) in a consanguineous family. Whole-exome sequencing revealed a novel, rare variant in the 5′ donor splice site of intron 32 of *ABCA5* that segregates with the phenotype in a homozygous recessive manner. *ABCA5* is highly expressed in human skin and hair follicles, and its expression pattern is conserved in mouse tissues as well. Importantly, the *ABCA5* loss-of-function mutation not only leads to a decrease in protein levels in both mesenchymal and epithelial compartments of CGHT patient hair follicles, but also to lysosomal dysfunction, which results in a defective clearance of autophagosomes under basal conditions and an overall accumulation of endolysosomal cholesterol in patient keratinocytes. We also identified an unrelated case of sporadic CGHT with a t3;17 translocation and cryptic 1.3 Mb deletion spanning *ABCA5*, and found that *ABCA5* levels were dramatically reduced in patient cells as well as throughout the hair follicle epithelium. Our findings implicate *ABCA5* as a gene with an essential role in hair growth.

## Results

### Clinical features, histology, and quantification of hair follicle length in congenital generalized hypertrichosis terminalis (CGHT)

**Figure 1:**
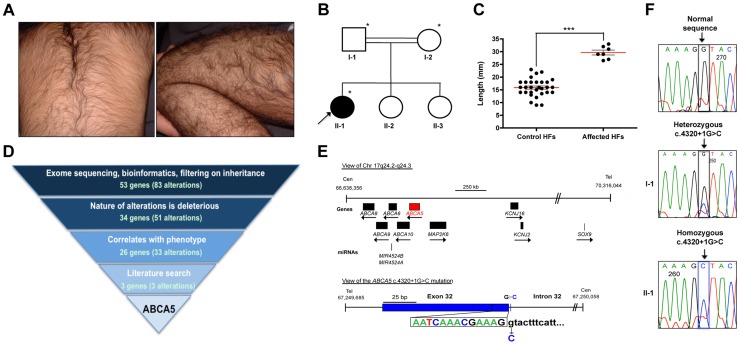
Whole-exome sequencing in a case of congenital generalized hypertrichosis terminalis (CGHT) revealed a splice site mutation in *ABCA5*. (A) Clinical photos of proband that illustrate the excessive hair overgrowth on the back (upper photo) and leg (lower photo). The proband is two years of age in this photo. (B) Pedigree of the family from a consanguineous union (indicated by double lines), where the proband (arrow) is the only affected member. Asterisks indicate the individuals on whom whole-exome sequencing was performed. (C) Quantification of the length (mm) of patient versus control hair follicles derived from the forearm revealed that patient hair follicles are 87% longer, with an average length of 29.6 mm (±0.9 mm) compared to 15.9 mm (±0.6 mm) for control follicles (***P<0.0001). (D) Whole-exome sequencing filtering strategy, including the number of alterations and genes obtained at each level. One alteration (c.4320+1G>C) was identified in the *ABCA5* gene, and cosegregation analysis in the family members revealed that both parents are carriers of this mutation, whereas the two unaffected sisters do not carry this mutation. (E) View of *ABCA5* (red) and the surrounding genes (black boxes) on chr17q24.2-q24.3, where arrows indicate direction of transcription. A magnified view of the c.4320+1G>C mutation in exon 32 of *ABCA5* with the exon 32-intron 32 boundary reference sequence annotated in colors corresponding to sequencing peaks. The G>C substitution is indicated below the control sequence. (F) Sequencing of control, carrier, and affected genomic DNA confirmed heterozygosity for the c.4320+1G>C mutation (boxed) in the father (I-1) and homozygosity in the proband (II-1). All coordinates reference the UCSC Genome Browser human reference genome hg19.

We ascertained a proband from Yemen with CGHT segregating with gingival hyperplasia as well as epilepsy. Excessive hair growth was observed on the face, including the forehead, cheeks, and upper cutaneous lip, arms, upper and lower back and legs (Figure 1A). No other members of the patient's family are affected, however, the parents of the proband were consanguineous, suggesting an autosomal recessive mode of inheritance (Figure 1B).

Analysis of patient hair follicles obtained from a whole skin biopsy of the forearm by hematoxylin and eosin staining demonstrated that the hairs are of the terminal type since they were medullated, pigmented and penetrated deep into the dermis (Figure S1). Patient hair follicles were thicker than those of controls, and anagen hairs were present in the skin biopsy, whereas no anagen hairs were detected in control skin biopsies. We measured the length of the hair shafts from plucked patient and control forearm hair follicles and found that the patient hair follicles were significantly longer (87%), with an average length of 29.6 mm (±0.9 mm) compared to 15.9 mm (±0.6 mm) for control follicles (*P<*0.0001) (Figure 1C).

### Whole-exome sequencing in CGHT identified a splice site mutation in *ABCA5*

To investigate the genetic basis of CGHT in this family, whole-exome sequencing was performed (Ambry Genetics) on genomic DNA obtained from the patient as well as both parents. Following sequencing, bioinformatics analysis (see Materials and Methods) and filtering on mode of inheritance ultimately lead to the identification of variants in 26 candidate genes (33 alterations) that were homozygous in the proband and heterozygous in both parents (Figure 1D). Further interpretative filtering based on literature searches focused on genotype-phenotype correlation revealed three candidate genes with homozygous mutations, *ABCA5, DGKZ,* and *ZNF253,* all of which are not currently associated with a Mendelian disease, and thus are considered novel. The nature of the homozygous mutations identified in these genes includes one splice site mutation (in *ABCA5:* c.4320+1G>C), one missense mutation (in *DGKZ:* c.1678C>T(p.P560S)), and one deletion (in *ZNF253:* c.429delA). Segregation analysis of these mutations revealed that both parents are heterozygous for all three mutations, whereas neither unaffected sister carries the *ABCA5* c.4320+1G>C and *DGKZ* c.1678C>T(p.P560S) mutations, and only one unaffected sister carries the *ZNF253* c.429delA mutation.

To determine whether these candidate genes are expressed in the skin and hair follicle, RT-PCR analysis was performed on RNA isolated from whole skin, which revealed that the *ABCA5* and *DGKZ* genes are abundantly expressed, suggesting a potential role for these two genes in the pathogenesis of the CGHT phenotype, whereas *ZNF253* is expressed at lower levels. The function of the *ZNF253* gene is unknown. The *DGKZ* gene encodes diacylglycerol (DAG) kinase zeta, a member of the DAG kinase family which phosphorylates DAG to phosphatidic acid and plays important roles in lipid signaling implicated in neurological diseases, including epilepsy, depression and Alzheimer's disease [11] – [13]. Moreover, mice deficient in the gene encoding DAG kinase, *epsilon (Dgke)* a member of the same gene family, exhibit features associated with epilepsy [14], suggesting the *DGKZ* substitution mutation may contribute to the pathogenesis of the patient's seizures. ABCA5, an ATP-binding cassette (ABC) protein, is a lipid transporter involved in the efflux of cellular cholesterol levels, and Abca5-deficient mice develop symptoms similar to several lysosomal diseases of the heart [15], [16]. Interestingly, *ABCA5* lies in the minimal common region to four reported familial cases and one sporadic case of autosomal dominant CGHT, both with and without gingival hyperplasia [4] – [6], suggesting that mutations in this gene may be associated with CGHT.

The *ABCA5* mutation results from a G-to-C substitution in the first nucleotide of intron 32 (Figure 1E). Sanger sequencing was performed on genomic DNA from the proband as well as the unaffected father, which confirmed homozygosity in the proband (II-2) and heterozygosity in the father (I-2) for the c.4320+1G>C mutation (Figure 1 E, F). Importantly, this mutation was not present in control individuals, determined by searching various genome databases and sequencing the genomic DNA of 10 control individuals. Moreover, a query for genetic variants that lie within the *ABCA5* locus using the UCSC Human Genome (hg19) and Ensemble Genome Browsers verified that this variant is not a SNP or common variant associated with any human genetic disease. Thus, the c.4320+1G>C mutation in *ABCA5* is novel.

In light of the cosegregation of the mutation with the disease phenotype in the family, association with the CGHT phenotype in previously reported cases, and reported expression in the human hair follicle with *ABCA5* mRNA levels being the highest out of the four genes within the minimal common region [6], we further investigated the consequence of this mutation on *ABCA5* mRNA splicing and the potential role for this protein in hair follicle growth.

### Sequencing of the mutant *ABCA5* transcript revealed aberrant splicing

**Figure 2:**
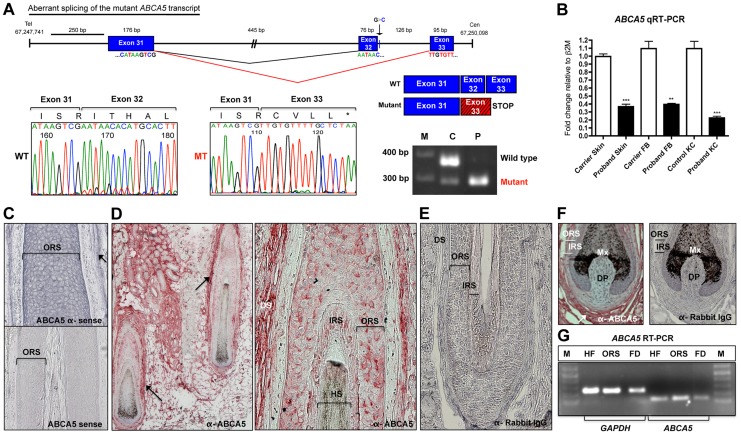
The *ABCA5* c.4320+1G>C mutation leads to aberrant splicing and nonsense mediated decay, and *ABCA5* is abundantly expressed in the skin and hair follicle. (A) Depiction of normal splicing between exons 31 and 32 (black lines) and aberrant skipping of exon 32 as a result of the c.4320+1G>C mutation (red lines), where the adjoining of exon 31 to 33 leads to premature termination during translation. The out-of-frame exon 33 sequence (red box) is indicated by the hatched lines, followed by a stop codon. Sequencing of the mutant transcript (MT) confirmed the aberrant splicing event, where the amino acid sequence is listed above and the stop codon is indicated by an asterisk. RT-PCR on RNA from whole skin followed by gel electrophoresis of the exon 31–33 amplicon demonstrates complete loss of the wild-type (WT) transcript in the patient (P), and very low levels of the mutant (MT) transcript in the carrier (C). (B) qRT-PCR of *ABCA5* transcript levels in the carrier and patient revealed a significant reduction of levels in patient whole skin, keratinocytes and fibroblasts. A Student unpaired t test was performed with a cutoff *P* value of 0.05 for statistical significance. (C) *ABCA5* is expressed at strong levels throughout the outer root sheath (ORS) and dermal sheath (arrow) of the human hair follicle by *in situ* hybridization using an antisense probe, whereas the sense probe produced minimal background signal. (D) ABCA5 localizes to the perifollicular dermis, dermal sheath, as well as the hair follicle ORS and inner root sheath (IRS) by immunohistochemistry on paraffin-embedded sections. (E) The anti-rabbit IgG primary antibody control produced no signal. (F) Immunohistochemistry of ABCA5 on hair follicle end bulbs revealed strong expression in the dermal sheath and perifolliclar dermis but no expression was observed in the dermal papilla. (G) *ABCA5* is endogenously expressed in plucked scalp hair follicles, microdissected ORS, as well as perifollicular dermis (including dermal sheath), determined by RT-PCR. Expression was normalized to *GAPDH* levels. M  =  marker.

*In silico* analysis of splicing events using the computational algorithms Berkley Drosophila Genome Project (BDGP) [17] and ESEfinder [18], [19] predicted that the c.4320+1G>C mutation results in complete loss of the donor splice site. To determine the consequence of the mutation at the transcript level, we amplified by RT-PCR the ∼200 bp region flanking the mutation from patient, carrier, and control mRNA. Sanger sequencing revealed that the mutation leads to aberrant skipping of exon 32 in the proband (Figure 2A). As a result of joining exon 31 to exon 33, the remainder of the transcript downstream of the mutation is out-of-frame, leading to a premature termination 14 bp downstream of the mutation (Figure 2A). RT-PCR analysis of the exon 31–33 amplicon using RNA isolated from whole skin revealed the complete absence of the wild-type transcript in the proband. In contrast, we found the presence of the wild-type transcript at high levels and the mutant transcript at very low levels in the father (Figure 2A). The mutant mRNA most likely undergoes nonsense-mediated decay, since the mutation resides near the 3′ end of the transcript and the aberrant splicing event is predicted to affect the overall stability of the mRNA.

To investigate this possibility, we compared the relative *ABCA5* mRNA levels between the proband and unaffected carrier father by qRT-PCR using primers located at the 5′ end of the mRNA, and found that transcript levels were significantly reduced in patient whole skin (2.7-fold; *p*<0.0001), cultured keratinocytes (2.8-fold; *p* = 0.0016), as well as fibroblasts (4.9-fold; *p*<0.0001), demonstrating that the mutant mRNA is unstable and undergoes nonsense-mediated decay (Figure 2B).

### ABCA5 is abundantly expressed in the epithelial and mesenchymal lineages of human and mouse hair follicles

While *ABCA5* expression was previously identified in plucked human hair follicles by RT-PCR analysis [6], the specific cell type(s) synthesizing *ABCA5* in the hair follicle and potential expression in the surrounding dermal tissue remained unknown. Using *in situ* hybridization on human hair follicles in the growth phase of the hair cycle (anagen), we observed *ABCA5* transcript expression in both the epithelial and mesenchymal compartments, present within the outer root sheath (ORS) of the hair follicle as well as dermal sheath (Figure 2C). To determine the localization pattern of ABCA5 protein in human skin and hair follicles, immunohistochemistry (IHC) was performed on paraffin-embedded skin sections and expression was most evident in the dermal sheath, perifollicular dermis, ORS, and IRS of hair follicles (Figure 2 D–F). Since the ABCA5 antibody is polyclonal, we validated endogenous *ABCA5* expression in the hair follicle as well as in the surrounding perifollicular dermis using RT-PCR on these tissues and observed strong *ABCA5* expression in plucked hair follicles (HF), microdissected ORS, as well as perifollicular dermis (FD) including the dermal sheath (Figure 2G).

To assess whether the human ABCA5 expression pattern was conserved in mouse skin and hair follicles, we first determined whether we could detect Abca5 immunoreactivity in a site of known *Abca5* expression using the same polyclonal antibody. In mice and rats, *Abca5* mRNA levels are abundant in the testis by Northern blotting and *in situ* hybridization [20], [21]. Using immunohistochemistry and immunofluorescence staining on adult mouse testis sections, we observed strong localization of Abca5 to the basal cells of the seminiferous tubules, interstitial cells consisting of Leydig cells (as previously reported in [21]), as well as the tunica albuginea (Figure S2 A–B, G). In the epididymis, we found very strong and specific localization of Abca5 to the connective tissue outlining the cylindrical epithelium in the corpus and cauda regions, including fibrocytes and smooth muscle cells, as well as within the basal and tall columnar cells of the corpus cylindrical epithelium (Figure S2 D–E, H–I). The control testis and epididymis sections (no primary antibody) yielded no signal (Figure S2 C, F). Importantly, we observed the same localization pattern of Abca5 in these tissues using two different fixatives; an organic solvent (methanol/acetone) and cross-linking agent (formalin), and our data are consistent with previous reports on *Abca5* mRNA expression [20], [21].

We next investigated the localization pattern of Abca5 in the mouse anagen hair follicle using immunofluorescence staining and immunohistochemistry, and observed high levels of Abca5 localization to the ORS and IRS of hair follicles (Figure S3). Expression was also observed in the dermal sheath and perifollicular dermis by the immunohistochemistry method (Figure S3 E–F), similar to what we observed in human hair follicles (Figure 2D). Importantly, ABCA5 localization in the skin and hair follicle appears to be conserved between human and mouse, and its broad expression pattern spans multiple cell lineages, both within and surrounding the hair follicle. Collectively, these data suggest a prominent, evolutionarily conserved role for this transporter in regulating hair growth.

### Homozygous loss-of-function of ABCA5, an *N-*glycosylated protein

**Figure 3:**
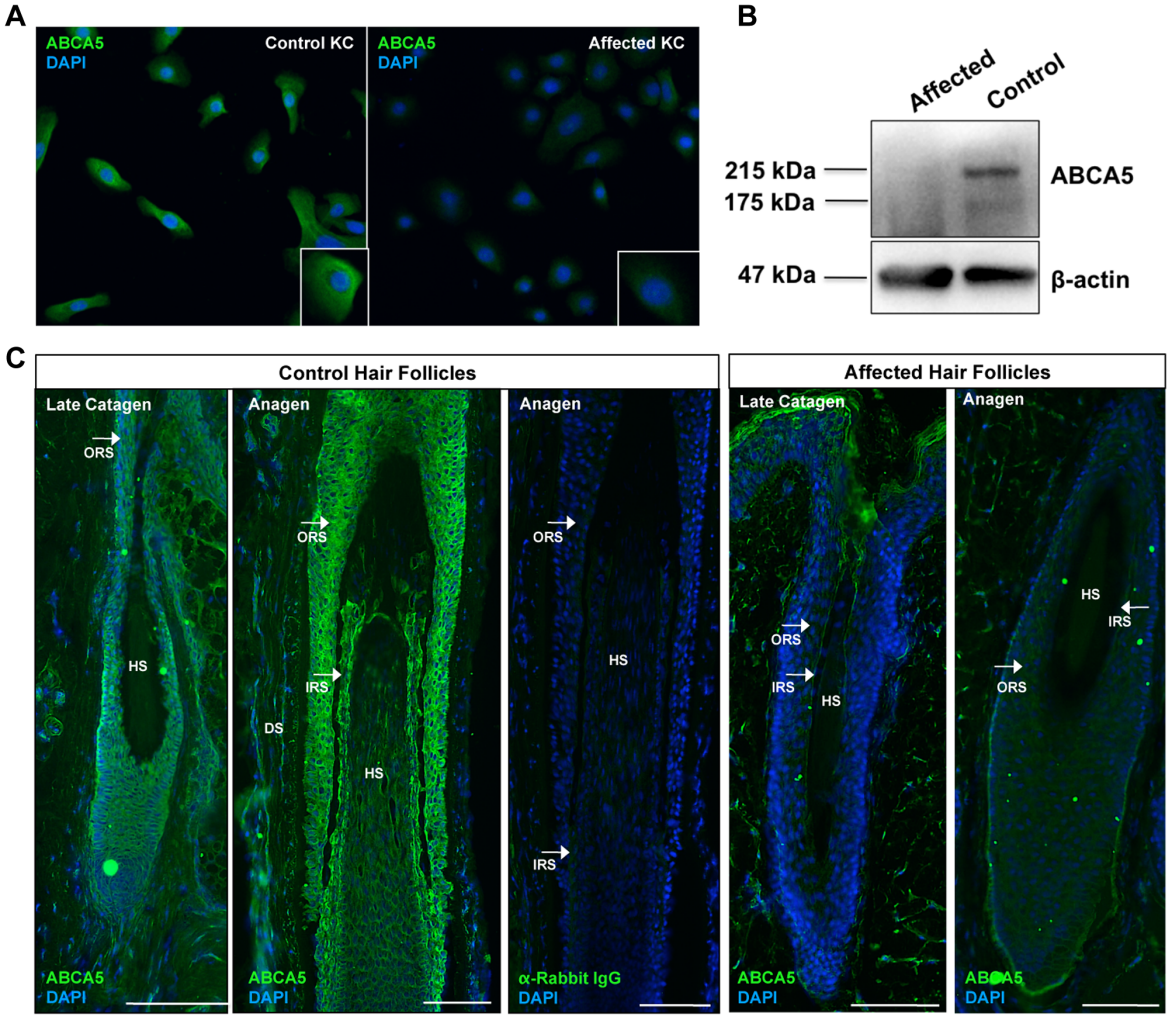
ABCA5 protein levels are significantly reduced in CGHT patient keratinocytes and hair follicles. (A) Immunofluorescence staining on cultured keratinocytes revealed a dramatic decrease in ABCA5 localization in CGHT keratinocytes compared to control (B) Immunoblotting on protein extracted from control and patient keratinocytes demonstrated loss of a 215 kDa band, which corresponds to the glycosylated form of the full-length ABCA5 protein, as well as a 187 kDa band, which is the unmodified protein in the patient compared to control. (C) Loss of ABCA5 localization to the outer root sheath (ORS) and inner root sheath (IRS) within patient hair follicles by immunofluorescence staining. Affected hair follicles in anagen and catagen were obtained from forearm skin biopsies, and control catagen hairs were obtained from forearm skin and anagen hairs from the occipital scalp. The anti-rabbit IgG primary antibody control produced no signal. HS  =  hair shaft; DP  =  dermal papilla; DS  =  dermal sheath.

To determine the consequence of the *ABCA5* c.4320+1G>C mutation at the protein level, we performed immunofluorescence staining on control and CGHT keratinocytes cultured from whole skin biopsies, and observed a striking reduction in ABCA5 localization (Figure 3A). Immunoblotting revealed the loss of a 215 kDa band corresponding to the full-length transporter in its glycosylated form, as well as a 187 kDa band, representing the unglycosylated form [15] (Figure 3B). While the full-length transporter was predominantly detected in keratinocytes, in fibroblasts, we observed the expression of a previously reported truncated variant [20] that produces a half-transporter and a ∼100 kDa band. Importantly, the band representing this truncated variant was not detectable in CGHT patient fibroblasts (Figure S4). Since ABCA5 is a reported glycoprotein possessing multiple sites for *N-*glycosylation, we investigated whether the c.4320+1G>C mutation abolished the glycosylated form of the protein in patient fibroblasts by incubating total protein in the presence or absence of the PNGaseF enzyme that removes all *N-*glycosyl modifications. Following immunoblotting, we observed that the band corresponding to the ∼100 kDa isoform that is absent in the proband represents the glycosylated form of the protein (Figure S4). Since glycosylation is an important post-translational modification crucial to the proper folding, stability, subcellular localization and/or even function of many lysosomal proteins, including ABC transporters [22], this finding suggests a loss-of-function of both the full- and half-transporters encoded by *ABCA5* in CGHT.

Lastly, to evaluate the consequence of the c.4320+1G>C mutation on ABCA5 protein localization at the tissue level, we performed immunofluorescence staining on patient and control hair follicles and observed a striking reduction of ABCA5 protein throughout the outer and inner root sheaths of patient hair follicles in catagen and anagen (Figure 3C). Importantly, loss of ABCA5 expression at the site of pathology for the phenotype further demonstrates that the c.4320+1G>C mutation results in a complete loss-of-function allele.

### ABCA5 loss-of-function in CGHT keratinocytes perturbs endolysosomal function

**Figure 4:**
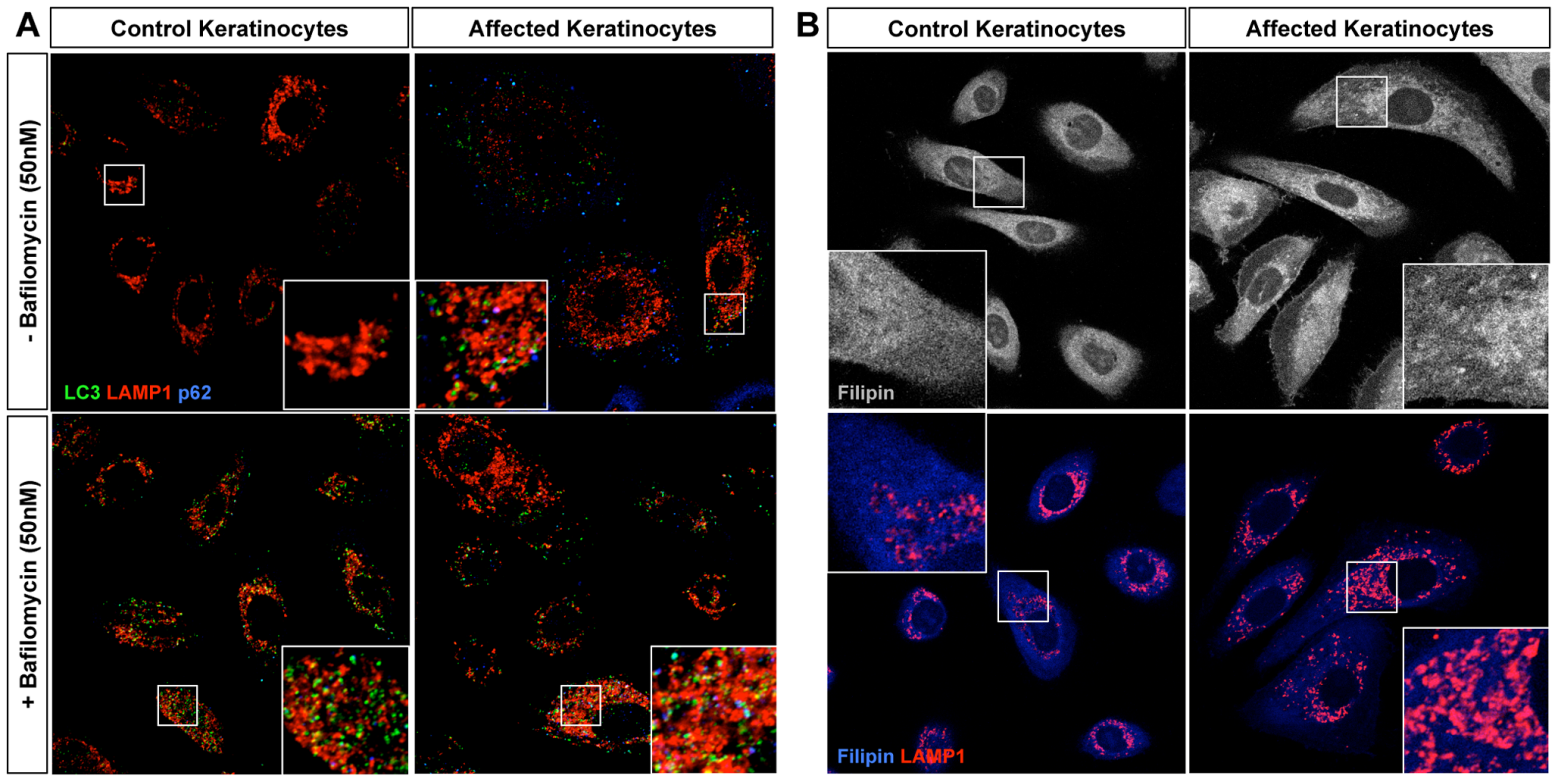
Homozygous loss-of-function of *ABCA5* perturbs lysosome function, resulting in an overall accumulation of autophagosomes and intracellular cholesterol levels in CGHT keratinocytes. (A) Control and CGHT keratinocytes were cultured in normal media in the presence or absence of 50 nM bafilomycin, fixed and immunostained. Confocal analysis of LC3 (green), Lamp1 (red) and p62 (blue) revealed an increased number of LC3-positive particles as well strong accumulation of p62 particles in affected keratinocytes. BAF-treated control cells possessed a two-fold increase in the number of LC3 puncta, whereas no significant difference was observed in BAF-treated affected cells (quantified in Figure S5). (B) Control and CGHT keratinocytes were fixed and immunostained for Filipin and Lamp1 (red), which revealed an accumulation and redistribution of free cholesterol to Lamp1-positive organelles in affected keratinocytes.

Lysosomes have been reported to play a role in hair cycling, where mice deficient for lysosomal proteases, cathepsin L (Ctsl) and lysosomal acid phosphatase 2 (Acp2), have delayed progression through the hair cycle resulting in periodic hair loss that is characterized by hyperproliferation of epithelial cells in the hair follicle and basal layer of the epidermis [23] – [26]. In mice, Abca5-deficient cells have aberrant processing of autolysosomes and autophagosomes [15], suggesting a role in lysosome integrity and/or autophagy, a catabolic process of intracellular digestion and recycling of organelles [27]. To gain insight into whether CGHT patient cells possessed intrinsic autolysosomal and/or autophagic defects, we visualized key markers of these organelles at the cytological level. LC3, a well-established marker for autophagosomes, is converted from its cytosolic form (LC3-I) to a lipidated form (LC3-II) which is able to re-localize and bind specifically to autophagosomal membranes [27]. A large pool of LC3-II is degraded upon lysosome-autophagosome fusion, when the internal content of autophagosomes is destroyed by lysosomal hydrolases. Immunofluorescence staining against endogenous LC3 revealed an increased number of LC3-positive particles (*i.e.*, autophagosome-like structures) in the affected keratinocytes compared with control, suggesting defects in the autophagy pathway (Figure 4A).

In order to discriminate between higher levels of basal autophagy and a defective clearance of autophagosomes, bafilomycin A1 (BAF), a proton pump inhibitor that blocks the acidification of lysosomes and thus the clearance of autophagosomes, was added to growing cells 2 hours prior to fixation. BAF treatment caused a 2-fold increase in the number of LC3-positive puncta representing autophagosomes and autophagolysosomes in control keratinocytes, compared to untreated keratinocytes (Figure 4A, Figure S5A). In contrast, BAF treatment failed to significantly increase the number of LC3-positive particles in the patient keratinocytes compared to untreated cells (Figure 4A, Figure S5A). Furthermore, immunofluorescence staining against p62, a polyubiquitin binding protein as well as an autophagic cargo, revealed a strong accumulation of puncta in patient keratinocytes, further suggesting autophagy defects (Figure 4A, Figure S5B). Overall, these results indicate that *ABCA5* loss-of-function in CGHT causes defects in the autophagy pathway, and more specifically, point to an impairment in the clearance of autophagosomes and autophagic flux under basal conditions. These results also suggest that the mechanism underlying the autophagy defects caused by the *ABCA5* mutation is a decrease in lysosomal function.

Because a previous report has shown a role for ABCA5 in the efflux of cholesterol in macrophages [16], we next tested whether patient cells exhibit defects in the metabolism and/or transport of free cholesterol. Additionally, because ABCA5 is localized to the lysosomal compartment, its mutation may affect the handling of lipoprotein-derived cholesterol in the endolysosomal system, perhaps contributing to the dysfunction of these organelles in patient cells. In order to visualize cholesterol, cells were stained with filipin, a polyene macrolide antibiotic and antifungal that fluoresces and detects unesterified free cholesterol [28]. Remarkably, the mutation in *ABCA5* produced an increase in intracellular filipin staining compared to controls (Figure 4B, top panel). More specifically, filipin-positive puncta were observed mostly inside Lamp1-positive structures (Figure 4B, lower panel) suggesting an accumulation of free cholesterol in the lumen of endolysosomal organelles, likely in their intraluminal vesicles. These data suggest that ABCA5 controls the fate of lipoprotein-derived cholesterol and that its mutation alters the intracellular traffic of free cholesterol, somewhat reminiscent of phenotypes observed in lysosome storage disorders, such as Niemann Pick disease Type C [29].

### Cytogenetic analyses and breakpoint mapping in a sporadic CGHT case revealed a t3;17 translocation and cryptic 1.3 Mb deletion encompassing *ABCA5*

At around the same time we identified the *ABCA5* c.4320+1G>C mutation in homozygous recessive CGHT, we independently studied this candidate region of chromosome 17 in a sporadic case of hypertrichosis. We ascertained a patient from Mexico with hypertrichosis universalis congenita (OMIM145700), whose parents (nonconsanguineous union) and siblings were unaffected. The patient exhibited excessive overgrowth of terminal hairs on the extremities, back, chest, and the face. Moreover, histological analysis of patient hair follicles obtained from a skin biopsy of the lower back revealed the presence of large anagen hair follicles that penetrate deep within the dermis (Figure S6).

**Figure 5:**
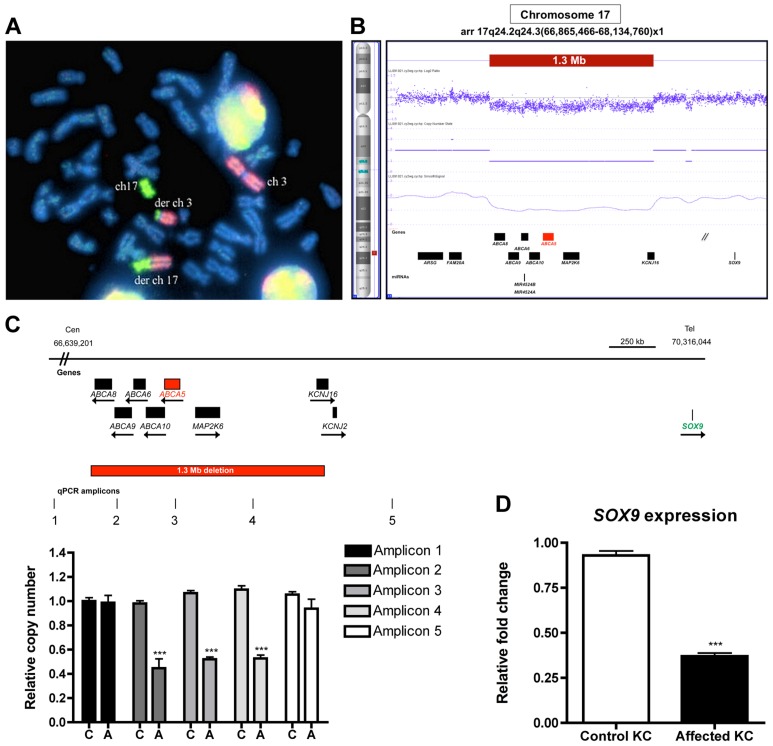
Cytogenetic analyses, breakpoint mapping, and copy number variant analysis in a sporadic case of CGH revealed a t3;17 translocation that leads to a cryptic 1.3 Mb deletion of chr17q24.2-24.3, and *SOX9* expression is reduced in patient keratinocytes. (A) Chromosomal paint was performed in a sporadic case of CGHT with commercially available whole chromosome probes for both chromosomes 3 and 17. Note: the probes do not bind to any of the other chromosomes except 3 and 17, and the derivative chromosomes suggesting that this is an isolated rearrangement between these two chromosomes. DAPI staining was used to visualize the entire chromosomal set (blue). (B) Copy number variant analysis using the Affymetrix Cytogenetics Whole Genome 2.7M array validated the cryptic deletion that spans 1.3 Mb (red box). RefSeq genes are depicted as black boxes. (C) Region of chr17q24.2-q24.3, including the genes and 1.3 Mb deletion identified by FISH and CNV analysis. The quantitative PCR (qPCR) amplicons are indicated as vertical lines, with three amplicons residing within and two flanking the deletion. qPCR was performed to confirm the deletion on control (C) and affected (A) genomic DNA using relative quantification and the primers listed in the Materials and Methods. (D) qRT-PCR of *SOX9* on RNA from control and affected keratinocytes revealed a 2.5-fold decrease (*P*>0.001) in expression in patient keratinocytes. A Student t test (unpaired) was performed with a cutoff *P* value of 0.05 for statistical significance. All coordinates reference the UCSC Genome Browser human reference genome hg19.

We initially performed karyotype analysis using G-banding of metaphase chromosomes that revealed a translocation between chromosomes 3q12 and 17q25, while the other chromosomes appeared cytogenetically normal. Chromosomal paint with two FISH probes against chromosomes 3 and 17 verified that no other chromosomes were involved in this rearrangement (Figure 5A), and telomere FISH (Figure S7A) confirmed that no subtelomeric sequences were lost as a result of the chromosome 17 breakpoint near the q telomere, suggesting an apparently balanced translocation event. To test whether this rearrangement segregated in the family, karyotype analysis was performed on both parents and siblings of the patient, but no abnormalities were detected, suggesting a *de novo* chromosomal rearrangement.

We next performed FISH analysis using BAC clones, which revealed a cryptic deletion at the breakpoint of chromosome 17, spanning BAC clones RP11-387O17 (chr17:66,354,485-66,568,819) and RP11-293K20 (chr17:67,076,187-67,261,438) (Figure S7B, C). To fine-map the deleted region, we utilized the Affymetrix 2.7M SNP array, which defined an 849.4 kb cryptic breakpoint deletion on chromosome 3p12.2 which did not contain any annotated genes, as well as a 1.3 Mb deletion on chromosome 17q24.2-q24.3 (Figure 5B) that encompassed 7 Ref Seq genes: members of the superfamily of ATP-binding cassette (ABC) transporters (*ABCA8, ABCA9, ABCA6, ABCA10, ABCA5*), mitogen-activated protein kinase kinase 6 (*MAP2K6*) and potassium inwardly-rectifying channel J16 (*KCNJ16*). We confirmed the 1.3 Mb deletion with quantitative PCR (qPCR) on patient and control genomic DNA using primers specifically designed to amplify sequences flanking and within the deleted region (Figure 5C). To determine whether the translocation event resulted in the disruption of a gene, we searched for transcripts that mapped to the vicinity of the breakpoints using the Genome Browser available at the UCSC database (hg19) and subsequently cloned the breakpoints, which did not reveal any evidence for gene disruption.

### The cryptic 1.3 Mb deletion at chr17q24.2-24.3 leads to a position effect on the downstream gene, *SOX9*

Several cases of CGHT have been reported in which CNVs on chr17q24.2-24.3 that lie within a 2.4 Mb region (Figure S8) have been identified and hypothesized to cause a position effect on the *SOX9* gene, a well-defined regulator of hair follicle stem cells [30], [31], which resides 1-2 Mb downstream of these variants. In our previous report of an autosomal dominant CGHT case, we found *SOX9* expression was dramatically reduced throughout the hair follicles of patients who possess duplications within the chr17q24.2-24.3 region [4]. To investigate the possibility that the 1.3 Mb deletion identified in the present study led to altered *SOX9* expression, we performed qRT-PCR on RNA isolated from patient and control keratinocytes and observed decreased *SOX9* expression in the patient keratinocytes by approximately 2.5-fold (*P*>0.001) (Figure 5D). This result is consistent with our previous findings, suggesting a contribution of decreased *SOX9* expression levels to the excessive hair overgrowth phenotype of CGHT. Given the association of SOX9 with CGHT and its known role in regulating hair follicle stem cells and outer root sheath differentiation [30], [31], we investigated the consequence of *ABCA5* loss-of-function on *SOX9* transcripts in the autosomal recessive CGHT case. Using qRT-PCR on patient and control keratinocytes, we did not detect a significant difference in *SOX9* gene expression (data not shown). While it remains possible that *SOX9* acts upstream of *ABCA5,* these genes may reside in separate pathways and utilize distinct mechanisms to contribute to the CGHT pathology.

### *ABCA5* mRNA and protein levels are dramatically reduced in both epithelial and mesenchymal compartments of the skin and hair follicle in sporadic CGHT

**Figure 6:**
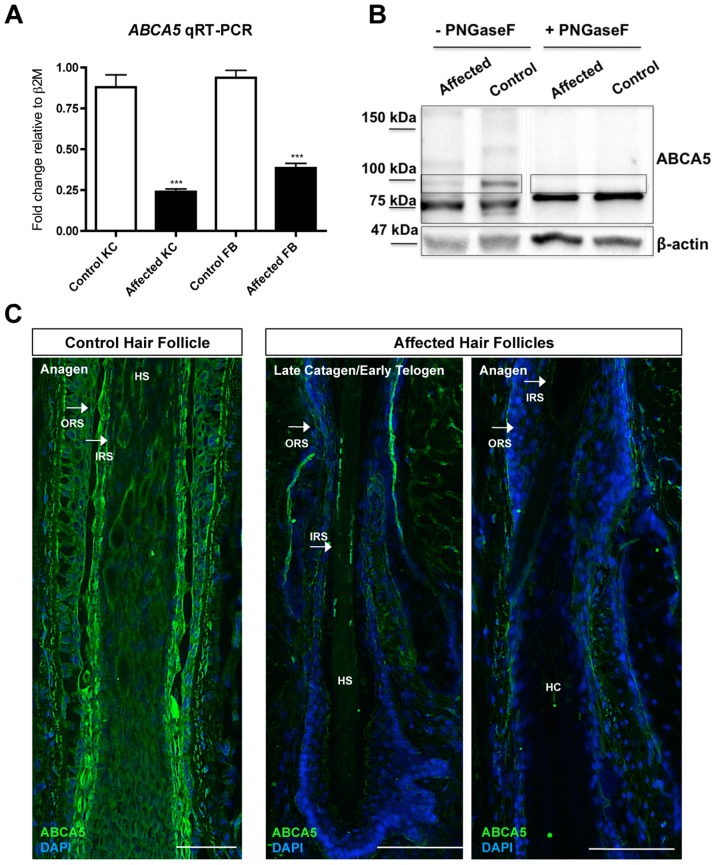
*ABCA5* levels are markedly reduced in patient hair follicles of the sporadic CGHT case. (A) qRT-PCR for *ABCA5* on control and affected keratinocytes and fibroblasts cultured from whole skin biopsies revealed a significant reduction in the patient cells compared to controls. A Student unpaired t test was performed with a cutoff *P* value of 0.05 for statistical significance. (B) Immunoblotting on protein extracted from carrier and patient fibroblasts in the presence or absence of the enzyme, PNGaseF revealed loss of a ∼100 kDa band that is the glycosylated form of the protein in patient fibroblasts relative to controls. (C) Immunofluorescence staining on control and patient hair follicles in the catagen and anagen stages demonstrated reduced ABCA5 localization throughout the outer root sheath (ORS) and inner root sheath (IRS) of patient hair follicles (compare to control catagen hair follicle in Figure 3C). HS  =  hair shaft; HC  =  hair canal.

Since the 1.3 Mb deletion we identified encompasses the *ABCA5* locus, and both sporadic and autosomal recessive CGHT patients possess excessive overgrowth of terminal hairs, we next investigated the possibility that the second allele in the sporadic CGHT case may harbor a mutation in the *ABCA5* gene. Therefore, we sequenced all the exons of *ABCA5* and ∼2 kb upstream of the gene, but did not find any mutations (data not shown). To test *ABCA5* mRNA expression levels in the sporadic CGHT case, we performed qRT-PCR in keratinocytes and fibroblasts cultured from a patient skin biopsy compared to control, and observed a 3.7-fold decrease in *ABCA5* transcript levels in patient keratinocytes (*p*<0.0001), and a 2.4-fold decrease in patient fibroblasts (*p*<0.0001) (Figure 6A). Immunoblotting in fibroblasts cultured from patient and control skin biopsies revealed a striking decrease in ABCA5 protein levels, and treatment with the *N-*glycosylase PNGaseF revealed loss of the glycosylated form of the protein (Figure 6B). Moreover, we found using immunofluorescence staining on patient and control hair follicles in catagen and anagen that ABCA5 protein levels were dramatically reduced throughout the outer and inner root sheath of the patient hair follicles (Figure 6C). Collectively, these results suggest that loss of one genomic copy of *ABCA5* and its surrounding regulatory elements severely disrupts expression from the other allele, and significantly reduces expression levels, suggesting haploinsufficiency of *ABCA5* in the sporadic case of CGHT.

## Discussion

In this study, we investigated the genetic basis of a case of CGHT using whole-exome sequencing and identified a homozygous recessive loss-of-function mutation in a donor splice site of *ABCA5* that cosegregates with the phenotype. The c.4320+1G>C mutation leads to loss of *ABCA5* expression and localization within patient keratinocytes, fibroblasts and hair follicles compared to controls. We found that endogenous *ABCA5* is highly expressed in both epithelial and mesenchymal compartments of the hair follicle, and that its homozygous recessive loss-of-function is associated with defective lysosomes, as well as an overall accumulation of autophagosomes and endolysosomal cholesterol. In a case of sporadic CGHT, we performed detailed cytogenetic analyses, breakpoint mapping, CNV analysis and expression studies, which revealed a 1.3 Mb deletion encompassing the *ABCA5* locus and markedly reduced *ABCA5* levels in patient cells and hair follicles.

In inherited hypertrichoses, the mechanism(s) that lead to an excessive hair overgrowth phenotype observed include an increased anagen duration, increased hair follicle density, as well as a vellus-to-terminal transformation, where fine, unpigmented and unmedullated hairs are stimulated to grow deeper into the dermis, widen, and become medullated as well as pigmented [1], [2]. In the case of CGHT, a condition that is present at birth, terminal hairs develop at sites of the body where vellus hairs should be, a developmental event that most likely occurs during late hair follicle morphogenesis or immediately following. Lanugo hairs shed *in utero* and then replaced by vellus hairs are instead replaced with terminal hairs. In addition to a vellus-to-terminal transformation, it is likely that the excessive hair overgrowth phenotype in CGHT is also contributed by an increased duration of anagen, as the hairs produced across the body are longer than what is considered normal for the age, race, and gender of the individual. In the autosomal recessive CGHT case in this study, we found that the length of hair follicles plucked from the forearm was significantly greater than that of control hair follicles. Moreover, histological analysis of patient skin biopsies from extensor skin of the extremities revealed the presence of large anagen hair follicles situated deep in the dermis, whereas hair follicles in anagen were not present in control skin biopsies.

Using several methods of detection, we found that ABCA5 is strongly expressed in both mesenchymal and epithelial lineages of the human hair follicle. In mouse hair follicles, we observed the same expression pattern as in human follicles, and using immunostaining with a polyclonal ABCA5 antibody, detected Abca5 localization in the testis and epididymis—positive sites of Abca5 mRNA expression. In the skin, the mesenchymal expression (perifollicular dermis, dermal sheath) appears more intense using formalin-fixed paraffin embedded (FFPE) samples, and the epithelial outer and inner root sheath expression is more intense using frozen sections. These differences are most likely artifacts of antigen accessibility and tissue preservation (organic solvent vs. cross-linking agent) between the two methods (i.e. dermal and subcutaneous tissue is better preserved in the FFPE sections). However, RT-PCR analysis and *in situ* hybridization demonstrated strong *ABCA5* expression in both epithelial and mesenchymal lineages, and negative controls for both mRNA and protein studies yielded no signal.

High levels of ABCA5 localization to the hair follicle IRS is intriguing, as several genes involving lipid metabolism or transport possess strong inner root sheath expression and have been described to underlie human genetic conditions with sparse hair. *P2RY5*, encoding an orphan G protein-coupled receptor, is mutated in patients with autosomal recessive wooly hair syndrome [32], [33]. Likewise, *LIPH* encodes the phospholipase A1 family member that converts phosphatidic acid into lysophosphatidic acid, and is mutated in patients with autosomal recessive wooly hair and hypotrichosis [32] – [34]. While the precise mechanism by which these genes control hair growth remains elusive, we postulate that the loss of a functional ABCA5 transporter in the IRS of patient hair follicles may contribute to excessive hair overgrowth through blocking cholesterol efflux.

Human *ABCA5* is also highly expressed in perifollicular dermal tissue, including the dermal sheath of the hair follicle, and the number of dermal papilla cells has been shown to be important in determining hair follicle size in mice [35]. Since patient hair follicles are larger and longer, we postulate that they may contain an increased number of dermal papilla cells, but were unable to directly test this using the skin biopsies we received. Additionally, the control of hair follicle size is linked to BMP levels in the skin, where mice overexpressing the BMP antagonist, Noggin, have enlarged anagen hair follicles [36]. Therefore, ABCA5 transport of cholesterol may normally limit hair growth and intersect with the BMP signaling pathway, such that in *ABCA5*-deficient hair follicles, the excess buildup of intra-endolysosomal cholesterol attenuates BMP signaling and recapitulates the enlarged hair follicle phenotype of the Noggin overexpression mouse model. Functional studies using *Abca5^-/-^* mice would be required to directly address this.

*ABCA5* has not previously been described to be associated with a human condition, particularly one manifesting a hair or skin phenotype. In humans, ABCA5 is a reported biomarker for tumor stem cells in osteosarcoma based on its overexpression, where it is also highly expressed in melanoma and undifferentiated colon and ovarian carcinomas [37] – [39]. Consistent with the reported role of ABC transporters in tumor biology conferring resistance to drugs and chemotherapy/cancer-related substrates *(i.e.* phospholipids and cholesterol) [40], *ABCA5* upregulation may protect undifferentiated tumor cells from an intracellular accumulation of cholesterol and other sterols.

Mutations in ABC transporters have been reported in several other genetic skin diseases, including *ABCA12* in lamellar ichthyosis type 2 as well as the severe and lethal ichthyosis, Harlequin ichthyosis, *ABCC9* in Cantú syndrome, and *ABCC6* in the connective tissue disorder pseudoxanthoma elasticum (PXE) [41] – [46]. *ABCA12* is expressed in the skin and its deficiency leads to abnormal lipid transport in keratinocytes, effectively compromising the skin barrier in a cell-autonomous manner [46]. Recently, dominant missense mutations have been reported in the ABC transporter gene, *ABCC9* in several cases of Cantú syndrome, a form of congenital hypertrichosis segregating with osteochondrodysplasia, distinctive facial features, as well as cardiac defects [44], [45]. The *ABCC9* gene encodes a transmembrane protein that is a constituent of a potassium channel complex, and electrophysiology experiments revealed that the missense mutations have gain-of-function or activating effects on the protein, as they reduce ATP-dependent inhibition of the channel [44]. Interestingly, the *ABCC6* gene encodes the multidrug resistance associated protein 6 (MRP6), and is exclusively expressed in the liver and kidney, suggesting that mutations lead to ectopic mineralization within the skin of PXE patients via a non cell-autonomous mechanism [47]. Importantly, this suggests that blocking the allocrite, or transported substrate, is the key determinant of the phenotype, because *ABCC6* itself is not expressed in the skin, yet its loss-of-function manifests with a cutaneous phenotype.

The allocrite of ABCA5 has remained unknown, however, we have observed a cholesterol transport defect in human CGHT keratinocytes and mouse Abca5 has previously been reported to play a role in cholesterol efflux in macrophages [16]. In rats and now mice, Abca5 localizes to the Leydig cells of the testis, a primary site for cholesterol processing and sterol hormone synthesis [21], suggesting cholesterol is a potential allocrite for this transporter. Importantly, these reports as well as the subcellular localization pattern of Abca5 are consistent with our observation that *ABCA5* deficiency in CGHT patient cells causes a redistribution of free cholesterol and accumulation of this sterol in the endolysosomal compartment. The resulting sequestration of cholesterol within these organelles may prevent the delivery of this essential lipid to other cellular compartments, such as the plasma membrane, altering their physical and signaling properties. Alternatively, accumulation of endolysosomal cholesterol may decrease the overall fitness of these organelles, leading to a decrease in the degradative capacity of lysosomes and causing secondary defects in autophagy.

Cholesterol as a covalent modification is required for the biological activity of several signaling proteins in the Wnt and Shh pathways that regulate hair follicle morphogenesis and cycling [48]. Overexpression and knockout mouse models for such molecules have demonstrated critical roles in hair follicle patterning and possess phenotypes reminiscent of hypertrichosis. For example, mice deficient for the *Smoothened (Smo)* gene in *Keratin-14*-expressing epithelia develop *de novo* and abnormally large hair follicles from increased mesenchymal SHH signaling [49], and the overexpression of a stabilized beta-catenin *(Ctnnb1)* induces early placode formation as well as an increased density of hair follicles [50]. We postulate that decreased free cholesterol in *ABCA5* deficient CGHT cells may modulate the range and/or morphogenetic activity of such proteins either in the epithelium or mesenchyme during hair follicle patterning. Future experiments employing lineage-specific deletions of ABCA transporters (i.e. *Abca1, Abca5*) will be useful in testing this, where free cholesterol levels can be measured biochemically and by filipin immunostaining, followed by a thorough analysis of hair follicle morphology and density. Similarly, a pharmacological approach to deplete sterols (i.e. cyclodextrin treatment) or block ABCA transporter activity (i.e. glyburide treatment) in pregnant dams during hair follicle morphogenesis may provide insight into the role of cholesterol transport in hair patterning and growth.

In mice, the biogenesis of cholesterol and other lipids has been shown to play a role in regulating hair growth, as mice deficient for *Stearyl-CoA Desaturase I (SCD1)*, the enzyme required for the biosynthesis of monounsaturated fatty acids that compose cholesterol esters as well as membrane phospholipids and triglycerides, develop alopecia and possess a sparse hair coat with dry, scaly skin [51] – [53]. Furthermore, topical treatment of mouse skin with a cholesterol biosynthesis inhibitor of a sterol precursor leads to hair loss through the induction of catagen [54]. It remains to be determined whether *Abca5^-/-^* mice possess a hair phenotype, since this has not been directly examined and hair phenotypes in mutant mice deficient for several factors can be subtle in the absence of detailed histological analysis [55]. If hair patterning is aberrant in *Abca5^-/-^* mice, lineage-specific deletions of *Abca5* followed by morphometric and densitometric analysis of hair follicles may provide insight into the target cell type underlying the pathology in CGHT.

In the sporadic CGHT case, we found that *ABCA5* transcripts were significantly reduced. One possible explanation for this is that a *trans* mechanism for regulating *ABCA5* gene expression via an enhancer or some other regulatory element is in place, such that deletion of one copy of *ABCA5* and its regulatory elements abrogates gene expression on the other allele. Conversely, *ABCA5* may be imprinted or expressed preferentially from one allele, in which case, deleting the transcriptionally-active allele would result in loss of gene expression, thereby recapitulating the phenotype of the loss-of-function mutation. Alternatively, *ABCA5* may be a dosage-sensitive gene, where altering the copy number is sufficient to produce a phenotype, but perhaps in a distinct manner from the loss-of-function mutation identified in the present study, since carriers are unaffected. Since we observed a decrease in *SOX9* expression in sporadic CGHT patient keratinocytes, consistent with our previous findings in an autosomal dominant CGHT case [4] and considering the known role of SOX9 in hair follicle stem cells [30], [31], it is likely that the excessive hair overgrowth phenotype observed in sporadic CGHT may be the result of reduced levels of both *ABCA5* and *SOX9* transcripts. We did not detect altered *SOX9* expression in the autosomal recessive CGHT case, suggesting either that *SOX9* acts upstream of *ABCA5* or that the two genes reside in separate pathways, employing distinct mechanisms to contribute to the CGHT pathology.

Importantly, this is the first report of the *ABCA5* gene to be associated with a human genetic condition, and the first reported case of CGHT in which a point mutation in a single gene contributes to the excessive hair overgrowth phenotype. Collectively, our findings provide insight into the mechanisms by which ABCA5 may directly or indirectly act to control hair growth at the subcellular level. While there is evidence to support a role for *ABCA5* in both epithelial and mesenchymal compartments of the hair follicle, the precise cell type(s) deficient for *ABCA5* that is associated with excessive hair overgrowth remains unclear. In light of our findings, we postulate that the loss-of-function of *ABCA5* during morphogenesis abrogates lysosome function and cholesterol efflux in hair follicle cells, leading to the aberrant activity of one or more downstream signaling pathways with crucial, defined roles in hair follicle induction, development and cycling.

## Materials and Methods

### Ethics statement

Informed consent was obtained from all subjects and approval for this study was provided by the Institutional Review Board of Columbia University in accordance with the Declaration of Helsinki Principles.

### Patient information and materials

In the autosomal recessive CGHT case, the proband is an eleven-year-old girl with congenital generalized hypertrichosis segregating with gingival hyperplasia and epilepsy. She was the product of a full-term uncomplicated pregnancy to a then 18-year old Yemeni mother whose husband is her first cousin. The patient's initial genetic evaluation was at 6 months of age; chromosome analysis revealed 46, XX normal complement and SNP arrays revealed multiple regions of homozygosity consistent with parental consanguinity. Severe hypertrichosis and a low anterior hairline that merged with the eyebrows were observed, and excessive body hair was present on the patient's back and extremities, as well as on the upper lip, genitalia, and axillary regions. Hair overgrowth progressed on her face trunk, eyebrows and eyelashes, and scalp as well as body hair is long and coarse. The gingival hyperplasia became evident at 18 months of age and progressed to the extent that it interfered with tooth eruption and feeding. Partial gum surgical resection was initially performed at 3 years of age and has been performed several additional times over the past eight years due to excessive gum overgrowth. Extensive endocrine and metabolic workups have been performed over the years and all results have been normal.

In the sporadic CGHT case, the patient initially presented to the clinic at the age of three months. On physical exam, she had universal hypertrichosis that was accentuated on the extremities, the back, chest, and the face. As expected, regions that are devoid of hair follicles including the palms, soles and distal phalanges were spared. In addition, the patient had unusual facial features, including a broad base of the nose, widely set eyes, and bulbous tip of the nose. The parents reported that the excessive body hair was shed during the first month of life, but then grew back with increased length. The mother did not report any drug intake, major illness or trauma during pregnancy, and the girl was born at full term. There is no history of hypertrichosis in the family. Extensive physical examination, radiological studies including X-rays (skull, long bones and hands), laboratory evaluations (complete blood count, blood chemistry, general urine examinations) and abdominal ultrasonography did not reveal any abnormalities.

In the autosomal recessive CGHT case, two small punch biopsies from extensor skin of the extremities, 3–5 cc of blood, in addition to plucked hairs from the forearm were received from the proband (II-1) and the unaffected father (I-1). For each individual, one biopsy was divided into two pieces: one flash frozen for RNA extraction and the other embedded in OCT for histological and immunological analyses. The other piece of whole skin was used for the culturing of keratinocytes and fibroblasts. In the sporadic CGHT case, a skin biopsy from the back of the affected individual was obtained; 3–5 cc of blood for DNA and RNA extraction was also obtained from the patient and her unaffected parents. Control hair follicles used in expression studies were obtained either from forearm skin biopsies or from occipital scalp biopsies, designated as non-human subject research under 45 CFR Part 46 and therefore IRB exempted for research.

### Whole-exome sequencing

Full exome sequencing, bioinformatics analysis (consisting of initial data processing, base calls, alignments, variant calls, nucleotide and amino acid conservation, biochemical nature of amino acid substitution, population frequency, and predicted functional impact), and filtering based on autosomal and X-linked dominant and recessive inheritance models was performed on the autosomal recessive CGHT case using genomic DNA from the proband (II-1), the unaffected mother (I-2), and the unaffected father (I-1) through Ambry Genetics (Allso Viejo, CA). Evaluation of relationships between the proband and unaffected parents was performed using Short Tandem Repeat markers and samples were prepared using the SureSelect Target Enrichment System (Agilent Technologies). In brief, genomic DNA was sheared, adaptor ligated, and PCR amplified, followed by incubation with the exome baits, elution, and then PCR amplification. Libraries were generated, quantified, and hybridized to the Illumina HiSeq 2000 flow cell for paired-end sequencing. Bioinformatics analysis was used for initial data processing, base calls, alignments as well as variant calls, and the Ambry Genetics Variant Analyzer tool (AVA) was used to determine conservation of nucleotide and amino acids as well as biochemical nature of substitutions, population frequency, and predicted functional impact. Various genome databases (Human Genome Mutation Database (HGMD), HapMap data, Single Nucleotide Polymorphism database (dbSNP), 1000 genomes) were used to search for previously described mutations and/or polymorphisms, and co-segregation studies were performed for candidate gene mutations. Full-exome sequencing revealed a total of 53 genes (85 alterations), and filtering based on the following criteria was used: deleterious nature of the alteration (34 genes, 5 unique alterations), removal of alterations clearly unrelated to patient's phenotype (26 genes, 33 alterations), and further interpretive analysis based on literature searches for genotype-phenotype correlation resulted in the selection of 3 genes (3 alterations) for further investigation.

### Nucleic acid extraction and sequencing

Genomic DNA was isolated from whole blood of the proband (I-1) and father (II-1) from the autosomal recessive CGHT case as well as from the patient with sporadic CGHT. Total RNA was extracted from forearm skin biopsies as well as from cultured keratinocytes and fibroblasts from family members I-1 and II-1 using the Qiagen RNeasy RNA extraction kit and standard methods.

PCR amplification of the exon 32-intron 32 boundary was performed in the autosomal recessive CGHT case using standard conditions and the following primers: F: 5′-GAACATCTTCAGAAGACTGTAAAG-3′ and R: 5′- GTAATCTGAGGATTCCCTAGCATAC -3′. To amplify and sequence the mutant *ABCA5* transcript, the following primers were used: F (in Exon 28): 5′-GCTGATGGGTTGCCAGTGTTGTGAAG-3′, and R (in Exon 33): 5′-CACATGTGCTGTTTGGCTTTGGGATC-3′. For sequencing of *ABCA5* in the sporadic CGHT case, 100–200 ng gDNA was used and primers were designed to flank the intron-exon boundaries 100–150 bp from each exon.

### Quantitative PCR on genomic DNA and quantitative RT-PCR on RNA

Quantitative PCR was performed on genomic DNA from the sporadic CGHT case to confirm the 1.3 Mb deletion identified; three amplicons on chromosome 17 were tested using Relative Quantification and the following primers: Amplicon 1 (chr17: 66639201-66639359, 159 bp): F: 5′-TAGATCATTCTCCTAAATGCTCTTCC -3′, R: 5′-GATGCAGCAAAGTTCTCAGGTG -3′; Amplicon 2 (chr17: 66958719- 66958932, 214 bp): F: 5′- GCTGAGCCTCTCCTGAAAACTGGACAAC -3′, R: 5′- GACTCAACTGACATAGGCCATGACAG -3′; Amplicon 3 (chr17: 67249799- 67249985, 213 bp): F: 5′- GAACATCTTCAGAAGACTGTAAAG -3′, R: 5′- CTGAGGATTCCCTAGCATACTTAGAGC -3′. Amplicon 4 (chr17: 67716661- 67716904, 244 bp): F: 5′- ACCATGTAAACAAGGAAAACAAC -3′, R: 5′- CTGAGGATTCCCTAGCATACTTAGAGC -3′. Amplicon 5 (chr17: 68403395- 68403416, 244 bp): F: 5′- CATTTATCCATATGGGAGGTAG -3′, R: 5′- AACAGATGTCCAAGAGAGTCAAATC -3′. Values were normalized to the *β2M* amplicon (161 bp) using the following primers: F: 5′- CACCTATCCCTGTTGTATTTTATCG -3′, R: 5′-CTCTTTATTTCTGCTGAGGTTTT-3′. All coordinates reference UCSC human reference genome build hg19.

Quantitative RT-PCR was performed on RNA isolated from whole skin, cultured keratinocytes and fibroblasts as previously described [5], [10] and as per the manufacturer's instructions. Relative quantification using the ddCT method [56] was performed with the *β2M* gene as the housekeeping control. The following primers were used for qRT-PCR assays: *ABCA5:* F: 5′- GAACCAACTTCAGGCCAGGTATT-3′, R: 5′-CACATGTGCTGTTTGGCTTTGGGATC -3′; *β2M:* F: 5′-GAGGCTATCCAGCGTACTCCA -3′, R: 5′- CGGCAGGCATACTCATCTTTT-3′; *SOX9:* F: 5′- AGTACCCGCACTTGCACAA-3′, R: 5′- CCGTTCTTCACCGACTTCCT-3′. *GAPDH:* F: 5′-GGAGCGAGATCCCTCCAAAAT -3′, R: 5′- GGCTGTTGTCATACTTCTCATGG-3′

### Statistical analysis

All experiments were performed in triplicates using materials from affected, carrier and control individuals. Experiments were repeated in triplicates, and a Student unpaired t-test was used to determine statistical significance in qPCR and qRT-PCR experiments with a *p* value of 0.05 as the cutoff value for significance. For autophagy experiments, quantification statistical analysis was performed using a two-tailed, equal variance Student's *t*-test. *P*-values of <0.05 (*), <0.01 (**), <0.001 (***) were determined to be statistically significant. The number of cells analyzed per condition is as follows: control, no bafilomycin: 61 cells; control, bafilomycin: 63 cells; affected, no bafilomycin: 37 cells; control, bafilomycin: 43 cells.

### Isolation and culturing of human keratinocytes and fibroblasts from whole-skin biopsies

Keratinocytes and fibroblasts were isolated and cultured from whole skin biopsies for the proband (I-1) and unaffected father (II-1) from the first case, and the patient from the second sporadic CGHT case using the method described in [10].

### *In situ* hybridization and immunohistochemical staining on whole skin

The DIG labeling system (Roche) was used to construct the sense and antisense riboprobes for *hABCA5* sequences, amplified using the following primers: *hABCA5* (Exon 13–19, 812 bp): F: 5′- GTGCAGAAGGTTTTACTAGATTTAGACA-3′, R: 5′- GTCTGGAACAAGTTTGATGGGAACCAC-3′; *hABCA5* (Exon 28–33, 550 bp): F: 5′-GCTGATGGGTTGCCAGTGTTGTGAAG-3′, R: 5′-CACATGTGCTGTTTGGCTTTGGGATC -3′. For human expression studies, *in situ* hybridization was performed on 10 µM skin and hair follicle sections from control, carrier and affected individuals using the methods described in [10].

Immunohistochemistry was performed on formalin-fixed paraffin-embedded (FFPE) sections of whole skin and hair follicles. In brief, slides were deparaffinized and rehydrated with a series of ethanol washes and then with 1X TBS. Antigen retrieval was performed for 10 minutes in a 1M sodium citrate, pH 6.0 solution heated to 95°C, and then slides were cooled and washed three times in 1X TBS. Tissues were blocked in either 10% normal donkey serum (Jackson ImmunoResearch, PA, USA) or 2% fish skin gelatin (Sigma Aldrich, MO, USA) and incubated with primary antibody in 1X TBS at 4°C overnight. The anti -rabbit ABCA5 antibody (ab99953, Abcam) was used at a concentration of 1:200 and the anti -rabbit IgG isotype primary antibody (Santa Cruz Biotechnologies, CA, USA) control was used at the same concentration. Slides were then washed with 1X TBS, incubated with the goat anti -rabbit biotin-conjugated antibody (1:800 in 1X TBS) for an hour at room temperature, washed again with 1X TBS and then incubated with the streptavidin-alkaline phosphatase (AP) tertiary antibody (Invitrogen; 1:300 in 1X TBS) for 30 minutes at room temperature. The SIGMA *FAST* Fast Red TR/Naphthol AS-MX Tablets (Sigma Aldrich, MO, USA) were used to develop the slides, which were then mounted with Dako Glycergel Mounting Medium (Dako, CA, USA).

### Immunofluorescence staining and immunoblotting

Immunofluorescence staining was performed on whole skin 10 µm sections from both human and mouse embedded in Optimal Cutting Temperature (O.C.T.). Slides were fixed with 50% MeOH/50% Acetone for 10 minutes at −20°C, washed with 1X PBS, and then blocked with 2% fish skin gelatin (Sigma Aldrich, MO, USA). The ABCA5 (Abcam, ab99953) and anti-rabbit IgG (Santa Cruz Biotechnologies) primary antibodies were used at a concentration of 1:200. Slides were then washed, incubated with the Alexa Fluor 488 donkey anti-rabbit IgG (Molecular Probes, Invitrogen) secondary antibody (1:800 in 1X PBS), mounted with VECTASHIELD mounting medium with DAPI (Vector Laboratories, Burlingame, CA, USA), and imaged using a LSM 5 laser-scanning Axio Observer Z1 confocal microscope (Carl Zeiss).

For autophagy analysis, human keratinocytes were grown to confluence, seeded on 12 mm coverslips, and then fixed with 4% paraformaldehyde for 20 min at room temperature. After permeabilization with 200 µg/ml digitonin (Invitrogen) in PBS for 10 min, cells were incubated with the specified primary antibodies for 1 hr at room temperature. Subsequently, cells were incubated with the appropriate Alexa-Fluor-conjugated secondary antibodies for 1 hr at room temperature, for cholesterol staining cells were additionally incubated with Filipin complex (Sigma) for 1 hr at room temperature. Images were acquired by confocal laser scanning microscopy (Zeiss LSM-700) and analyzed with Zeiss Zen and ImageJ Software (NIH). The number of LC3-positive compartments and their surface areas (expressed as number of pixels per field) were normalized to the number of cells in each field. The average size was obtained by dividing the surface area of the LC3-positive compartment (in pixel^2^) by the number of LC3 puncta. Similar measurements were made for Lamp1 and p62 compartments. Primary antibodies used for immunofluorescence: mouse anti-LC3 (MBL), guinea pig anti-p62 (Progen), rabbit anti-Lamp1 (Abcam) mouse anti-Lamp2 (Santa Cruz). Bafilomycin A1 (50 nM, Wako) was added to the media 2 hours prior to cell fixation.

Immunoblotting was performed using 10 µg total protein extracted with RIPA buffer/proteinase inhibitor cocktail from cultured keratinocytes and fibroblasts and SDS-PAGE was used to separate proteins, followed by a wet transfer to a hybond ECL nitrocellulose (Amersham, NJ, USA) or PVDF membrane (BioRad, CA, USA) membrane. All membranes were blocked with 5% milk for one hour at room temperature and then incubated with the primary antibodies, ABCA5 (1:500) and beta-actin (1:1000; Santa Cruz Biotechnologies, CA, USA) diluted in Washing Buffer (1X PBS 0.1% Tween-20). Membranes were then washed several times in 15-minute intervals with 1X PBST, incubated with goat anti-rabbit or mouse- HRP conjugated secondary antibody (Invitrogen; 1:1000), and then developed with the SuperSignal West Dura Extended Duration Substrate (Thermo Scientific, IL, USA).

### Cytogenetic analyses

G-banding analysis was performed using standard techniques, and FISH using chromosomal paint was performed on metaphase chromosomes obtained from peripheral blood leukocytes (PBLs), as per the manufacturer's directions (VYSIS). Sub-telomeric FISH was performed using a mixture of probes for chromosomes 17q, 17centromere, 9p, and 9q obtained from VYSIS in accordance with the manufacturer's instructions. Chromosomes were counterstained with DAPI (VYSIS) and hybridized metaphase chromosomes were viewed using a Nikon microscope fitted with a filter wheel and Cytovision Applied Imaging software.

### Breakpoint mapping and copy number variant (CNV) analysis

Breakpoint mapping was performed using FISH with BAC clones in the chromosome 17q24 region. To fine map the deleted region on chromosome 17q, genome scanning was initially performed using the Affymetrix 500k whole-genome mapping array, and then the 2.7M array (Affymetrix) to further examine CNVs in the region of the breakpoint.

## Supporting Information

Figure S1 Histological analysis of CGHT and control hair follicles by hematoxylin and eosin staining. (A–C) Hematoxylin and eosin staining of a patient skin biopsy from the forearm demonstrated that hair follicles are of the terminal type, as they are medullated, pigmented, and penetrate deep in the dermis. (B) Enlarged image of a late catagen hair follicle. (C) Magnified image of an anagen hair follicle situated deep in the dermis (A). Note the thickness of the outer root sheath compared to control hair follicles (D–F). (D) Hematoxylin and eosin staining of a control skin biopsy from the forearm. (E) Magnified image of a hair in catagen. (F) Enlarged image of the apoptosing strand of the catagen hair follicle. Note the size of the control hair follicle compared to patient hair follicles. No anagen hair follicles were present in the control skin biopsy. DS  =  dermal sheath; PFD  =  perifollicular dermis; IRS  =  inner root sheath; ORS  =  outer root sheath; HS  =  hair shaft; SG  =  sebaceous gland; DP  =  dermal papilla.
(TIF)

Figure S2 Mouse Abca5 localization pattern in the adult testis and epididymis by immunohistochemistry and immunofluorescence staining. (A, B) Immunohistochemistry on formalin-fixed paraffin-embedded (FFPE) adult mouse testis demonstrated strong localization of Abca5 to the basal cells of the seminiferous tubules (arrows in (A)), interstitial space consisting of Leydig cells, and tunica albuginea (arrow in (B)). (D, E) Abca5 immunohistochemistry on formalin-fixed paraffin-embedded adult mouse epididymis revealed strong localization to the connective tissue, smooth muscle cells and fibrocytes surrounding the cylindrical epithelium within the corpus and cauda regions, as well as within the basal and tall columnar cells of the cauda cylindrical epithelium (E). (C, F) Testis and epididymis sections incubated without primary antibody produced no signal. (G–I) Immunofluorescence staining of Abca5 in the testis and epididymis demonstrated Abca5 localization to the same structures within the testis and epididymis as did the immunohistochemical staining method.
(TIF)

Figure S3 Mouse Abca5 localizes to the outer and inner root sheath of anagen hair follicles. (A, B) Immunofluorescence staining of Abca5 on frozen mouse anagen skin (day 30) sections demonstrated a signal only within hair follicles (A), specifically within the outer and inner root sheath (B, C). (E–F) Immunohistochemical staining on formalin-fixed paraffin-embedded (FFPE) anagen skin sections revealed Abca5 localization to the outer and inner root sheath, and some signal present in the follicular dermis including the dermal sheath. No signal was observed on sections incubated without primary antibody (D, G).
(TIF)

Figure S4 Immunoblotting on protein extracted from carrier and patient fibroblasts in the presence of absence of the enzyme, PNGaseF that removes all *N-*glycosyl modifications revealed loss of a ∼100 kDa band that is the glycosylated form of the protein in the patient relative to the carrier. β-actin was used as a loading control.
(TIF)

Figure S5 Quantification of immunofluorescence staining for LC3 and p62 reveals defective autophagic clearance in CGHT. (A) The formation of LC3 puncta was significantly increased in affected vs. control keratinocytes (p<0.05, – Bafilomycin; p<0.01, + Bafilomycin) as well as within control keratinocytes + Bafilomycin vs. – Bafilomycin treatment (p<0.01), but no significant difference was observed between affected keratinocytes + Bafilomycin vs. – Bafilomycin treatment. (B) The formation of p62 puncta was significantly increased in affected vs. control keratinocytes (p<0.01) as well as within control keratinocytes + Bafilomycin vs. – Bafilomycin treatment (p<0.01), but no significant difference was observed between affected keratinocytes + Bafilomycin vs. – Bafilomycin treatment. A Student t test (unpaired) was performed with a cutoff *P* value of 0.05 for statistical significance and error bars represent the standard deviation. ImageJ was used for image quantification.
(TIF)

Figure S6 Histological analysis of hair follicles from the sporadic CGHT case**.** Hematoxylin and eosin staining of a patient skin biopsy from the lower back reveals the presence of terminal hair follicles in the anagen stage. DS  =  dermal sheath; PFD  =  perifollicular dermis; IRS  =  inner root sheath; ORS  =  outer root sheath; HC  =  hair canal; SG  =  sebaceous gland.
(TIF)

Figure S7 Telomere FISH and FISH using BAC clones spanning chromosome 17q24.2-24.3 to detect the 1.3 Mb cryptic deletion in sporadic CGHT**.** (A) Telomere FISH was performed to test possible deletions at the end of chromosome 17q using a commercially available probe mix for 17q (green), 17 centromere (green), 9p (green), and 9q (red). Note the presence of the green signal on the derived chromosome 3 indicating that the telomere of chromosome 17 was not deleted in the t3;17 rearrangement. (B–C) FISH using BAC clones on chromosome 17q24.2-q24.3 revealed a cryptic deletion at the breakpoint of chromosome 17. Metaphase spreads and interphase nuclei show only one signal for clones RP11-387O17 (green) (B) and RP11-293K20 (green) (C), which hybridize to the deleted 1.3 Mb portion of chromosome 17q24.2-24.3.
(TIF)

Figure S8 Summary of CNVs within the chr17q24.2-24.3 region identified in autosomal dominant and sporadic cases of CGHT illustrates that ABCA5 is located in the minimal common region. *ABCA5* (red box) and the other genes in the surrounding region (black boxes) as well as direction of transcription (arrows) are indicated. Nature of the variants (duplications, deletions) previously reported as well as identified in this study is indicated as well as the sizes and corresponding references. qPCR amplicons are represented by vertical lines, where two amplicons flank the 1.3 Mb deleted region (red box) and three amplicons lie within it. Database of Genomic Variants (DGV) alterations are indicated as gray boxes. All variants lie 1–2 Mb upstream of the *SOX9* gene. All coordinates reference the UCSC Genome Browser human reference genome hg19.
(TIF)
