# Supplementary figures and images for: Mutations in the Cholesterol Transporter Gene ABCA5 Are Associated with Excessive Hair Overgrowth
Source: PLoS Genet. 2014 May 15;10(5):e1004333. doi: 10.1371/journal.pgen.1004333 (PMC4022463; doi:10.1371/journal.pgen.1004333)

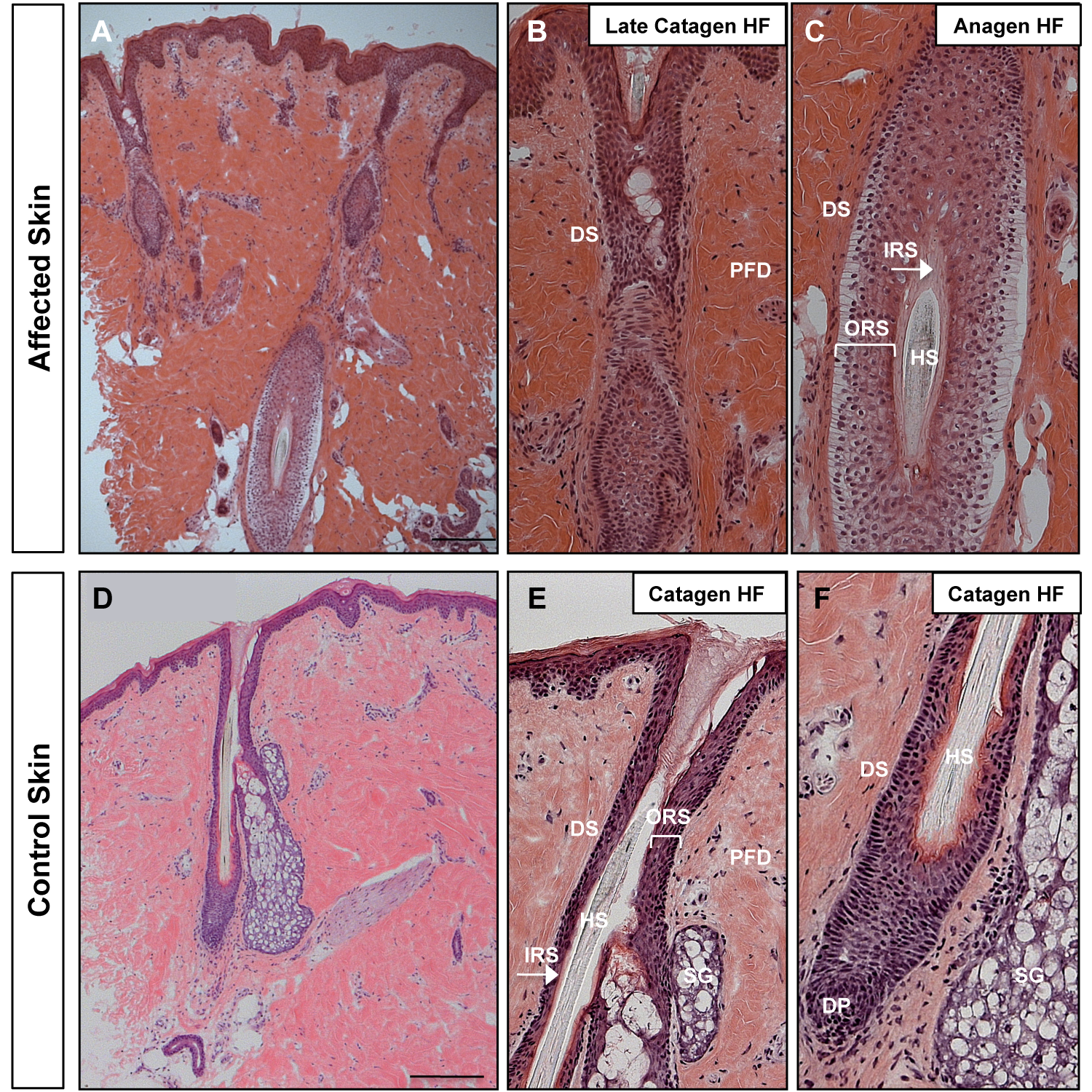

Supplement: Figure S1 — Histological analysis of CGHT and control hair follicles by hematoxylin and eosin staining. (A–C) Hematoxylin and eosin staining of a patient skin biopsy from the forearm demonstrated that hair follicles are of the terminal type, as they are medullated, pigmented, and penetrate deep in the dermis. (B) Enlarged image of a late catagen hair follicle. (C) Magnified image of an anagen hair follicle situated deep in the dermis (A). Note the thickness of the outer root sheath compared to control hair follicles (D–F). (D) Hematoxylin and eosin staining of a control skin biopsy from the forearm. (E) Magnified image of a hair in catagen. (F) Enlarged image of the apoptosing strand of the catagen hair follicle. Note the size of the control hair follicle compared to patient hair follicles. No anagen hair follicles were present in the control skin biopsy. DS = dermal sheath; PFD = perifollicular dermis; IRS = inner root sheath; ORS = outer root sheath; HS = hair shaft; SG = sebaceous gland; DP = dermal papilla. (TIF) [file pgen.1004333.s001.tif]

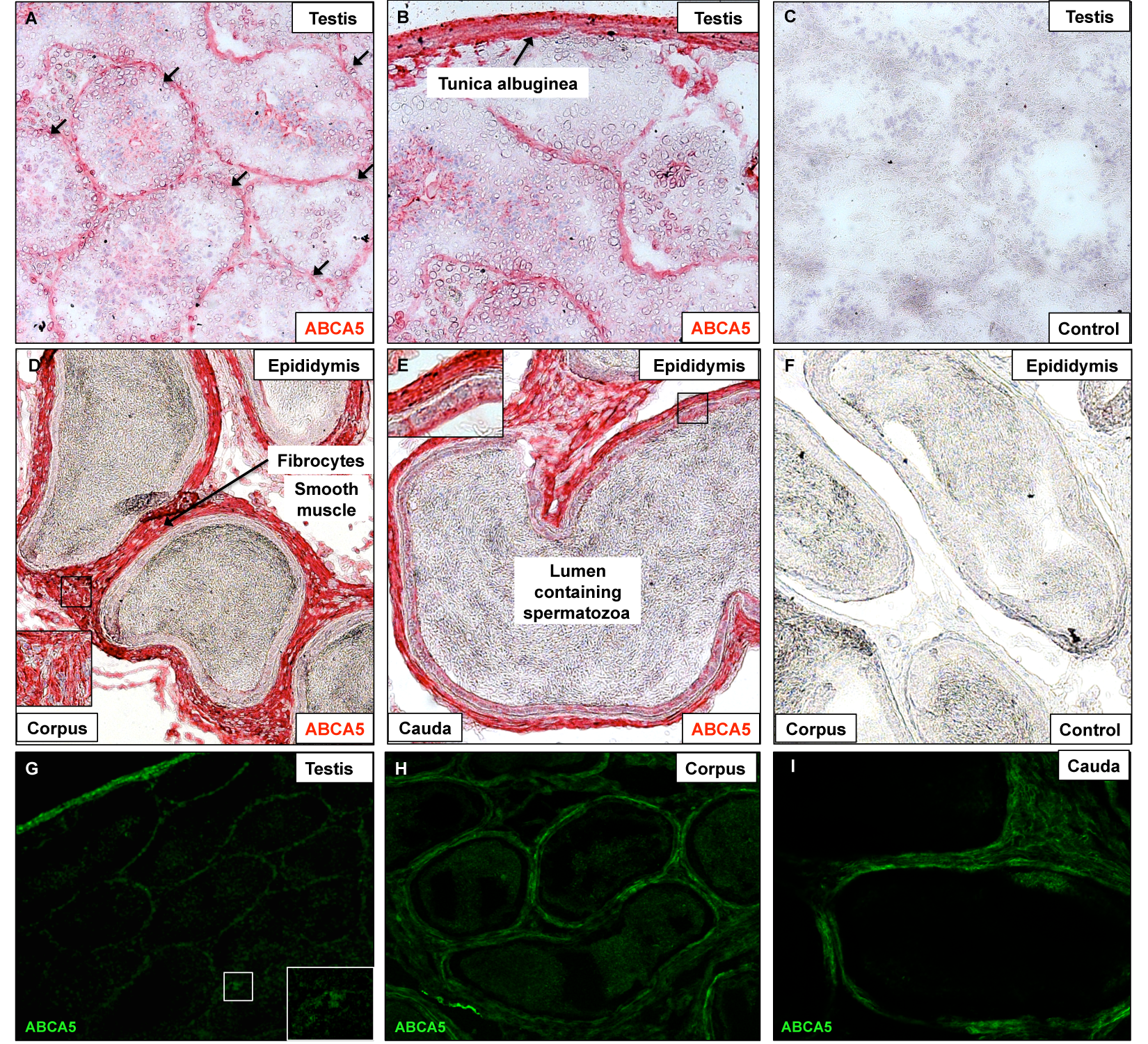

Supplement: Figure S2 — Mouse Abca5 localization pattern in the adult testis and epididymis by immunohistochemistry and immunofluorescence staining. (A, B) Immunohistochemistry on formalin-fixed paraffin-embedded (FFPE) adult mouse testis demonstrated strong localization of Abca5 to the basal cells of the seminiferous tubules (arrows in (A)), interstitial space consisting of Leydig cells, and tunica albuginea (arrow in (B)). (D, E) Abca5 immunohistochemistry on formalin-fixed paraffin-embedded adult mouse epididymis revealed strong localization to the connective tissue, smooth muscle cells and fibrocytes surrounding the cylindrical epithelium within the corpus and cauda regions, as well as within the basal and tall columnar cells of the cauda cylindrical epithelium (E). (C, F) Testis and epididymis sections incubated without primary antibody produced no signal. (G–I) Immunofluorescence staining of Abca5 in the testis and epididymis demonstrated Abca5 localization to the same structures within the testis and epididymis as did the immunohistochemical staining method. (TIF) [file pgen.1004333.s002.tif]

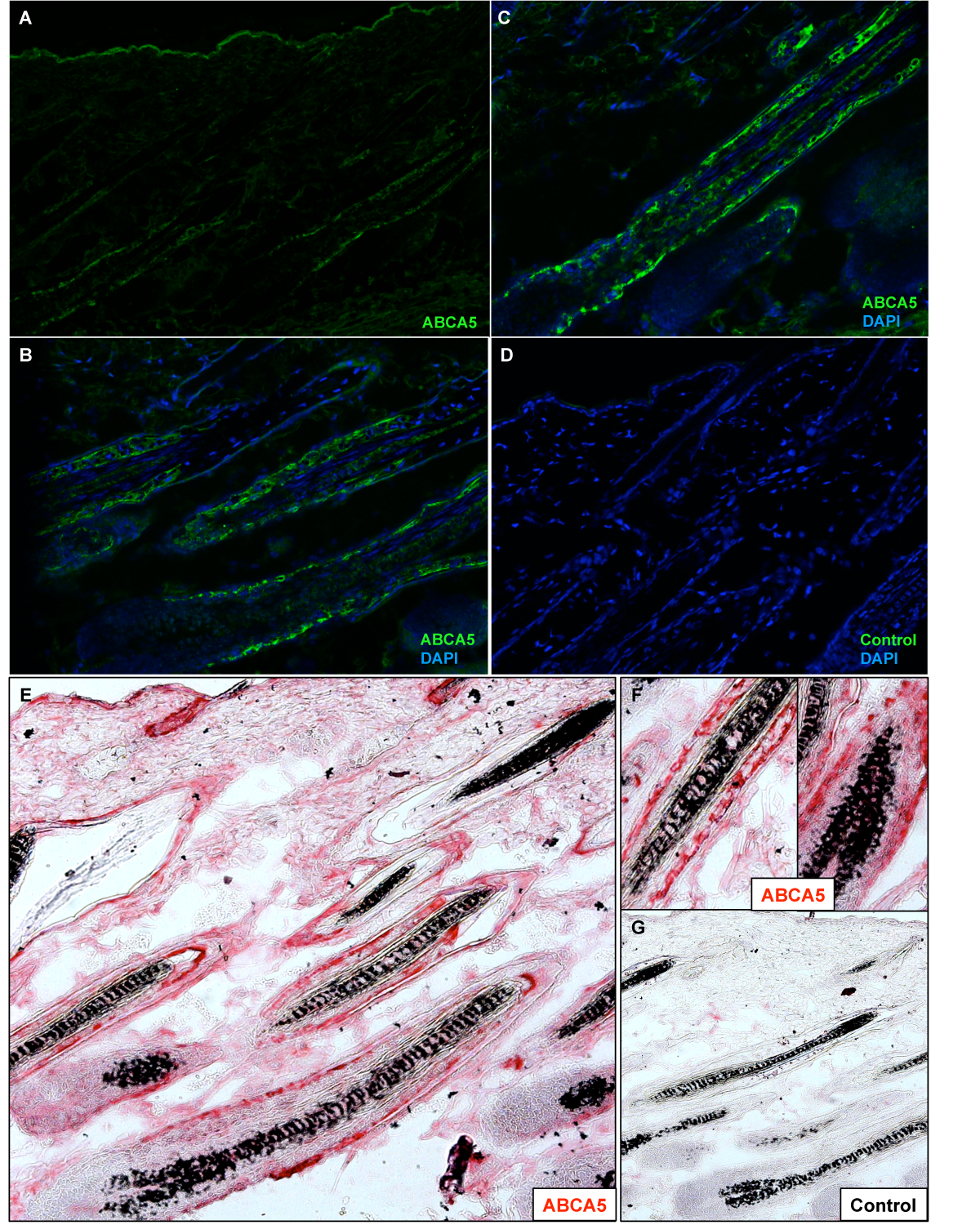

Supplement: Figure S3 — Mouse Abca5 localizes to the outer and inner root sheath of anagen hair follicles. (A, B) Immunofluorescence staining of Abca5 on frozen mouse anagen skin (day 30) sections demonstrated a signal only within hair follicles (A), specifically within the outer and inner root sheath (B, C). (E–F) Immunohistochemical staining on formalin-fixed paraffin-embedded (FFPE) anagen skin sections revealed Abca5 localization to the outer and inner root sheath, and some signal present in the follicular dermis including the dermal sheath. No signal was observed on sections incubated without primary antibody (D, G). (TIF) [file pgen.1004333.s003.tif]

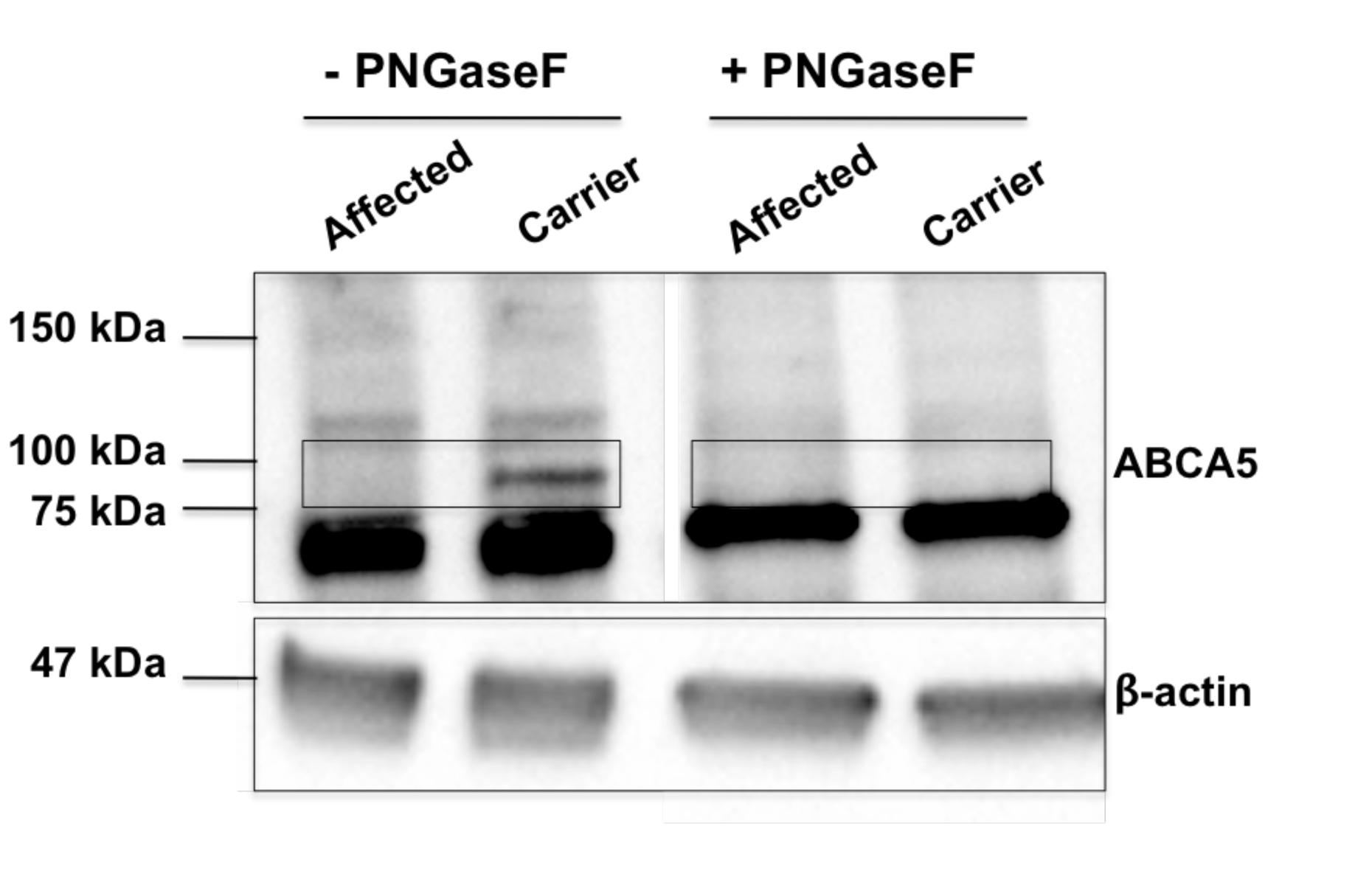

Supplement: Figure S4 — Immunoblotting on protein extracted from carrier and patient fibroblasts in the presence of absence of the enzyme, PNGaseF that removes all N-glycosyl modifications revealed loss of a ∼100 kDa band that is the glycosylated form of the protein in the patient relative to the carrier. β-actin was used as a loading control. (TIF) [file pgen.1004333.s004.tif]

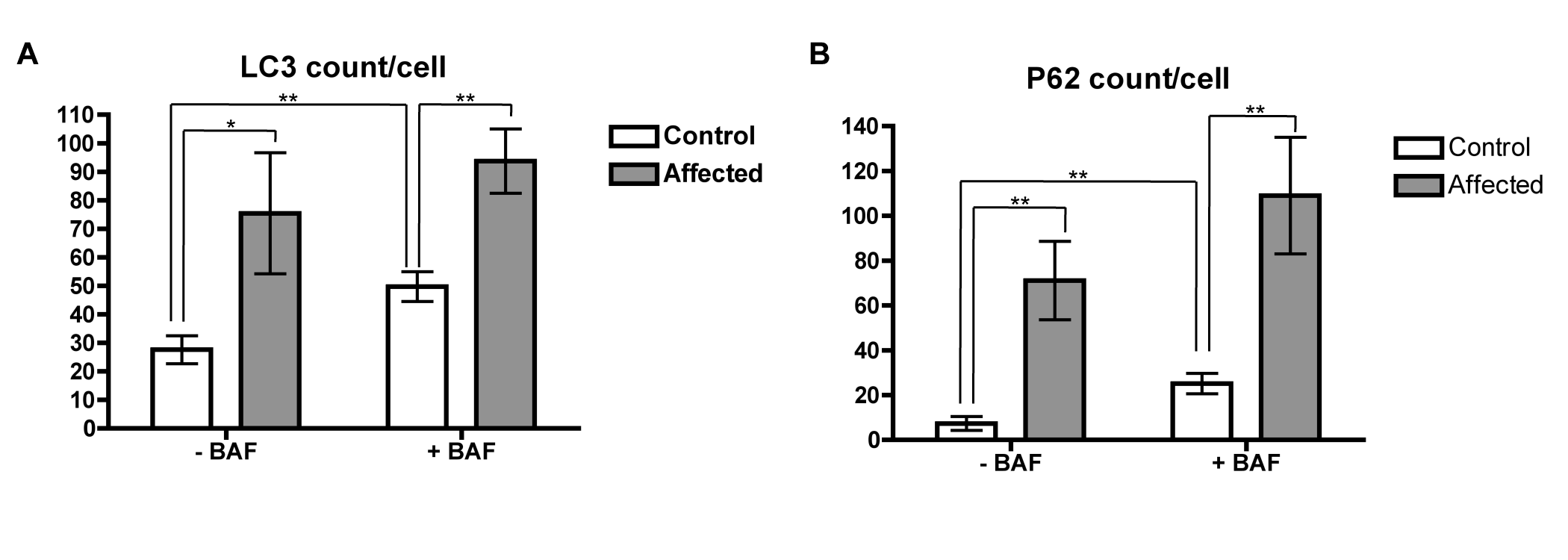

Supplement: Figure S5 — Quantification of immunofluorescence staining for LC3 and p62 reveals defective autophagic clearance in CGHT. (A) The formation of LC3 puncta was significantly increased in affected vs. control keratinocytes (p<0.05, – Bafilomycin; p<0.01, + Bafilomycin) as well as within control keratinocytes + Bafilomycin vs. – Bafilomycin treatment (p<0.01), but no significant difference was observed between affected keratinocytes + Bafilomycin vs. – Bafilomycin treatment. (B) The formation of p62 puncta was significantly increased in affected vs. control keratinocytes (p<0.01) as well as within control keratinocytes + Bafilomycin vs. – Bafilomycin treatment (p<0.01), but no significant difference was observed between affected keratinocytes + Bafilomycin vs. – Bafilomycin treatment. A Student t test (unpaired) was performed with a cutoff P value of 0.05 for statistical significance and error bars represent the standard deviation. ImageJ was used for image quantification. (TIF) [file pgen.1004333.s005.tif]

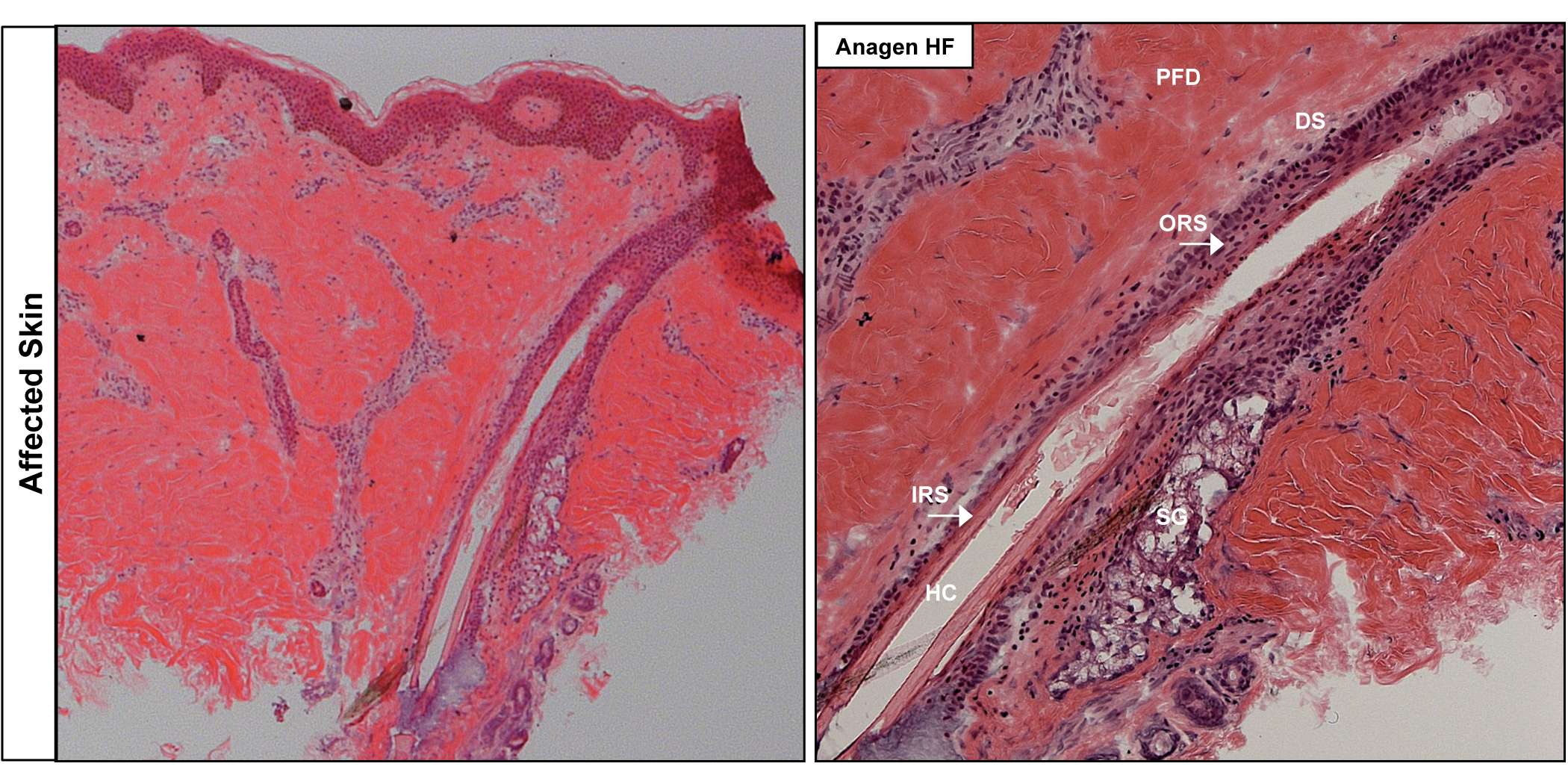

Supplement: Figure S6 — Histological analysis of hair follicles from the sporadic CGHT case. Hematoxylin and eosin staining of a patient skin biopsy from the lower back reveals the presence of terminal hair follicles in the anagen stage. DS = dermal sheath; PFD = perifollicular dermis; IRS = inner root sheath; ORS = outer root sheath; HC = hair canal; SG = sebaceous gland. (TIF) [file pgen.1004333.s006.tif]

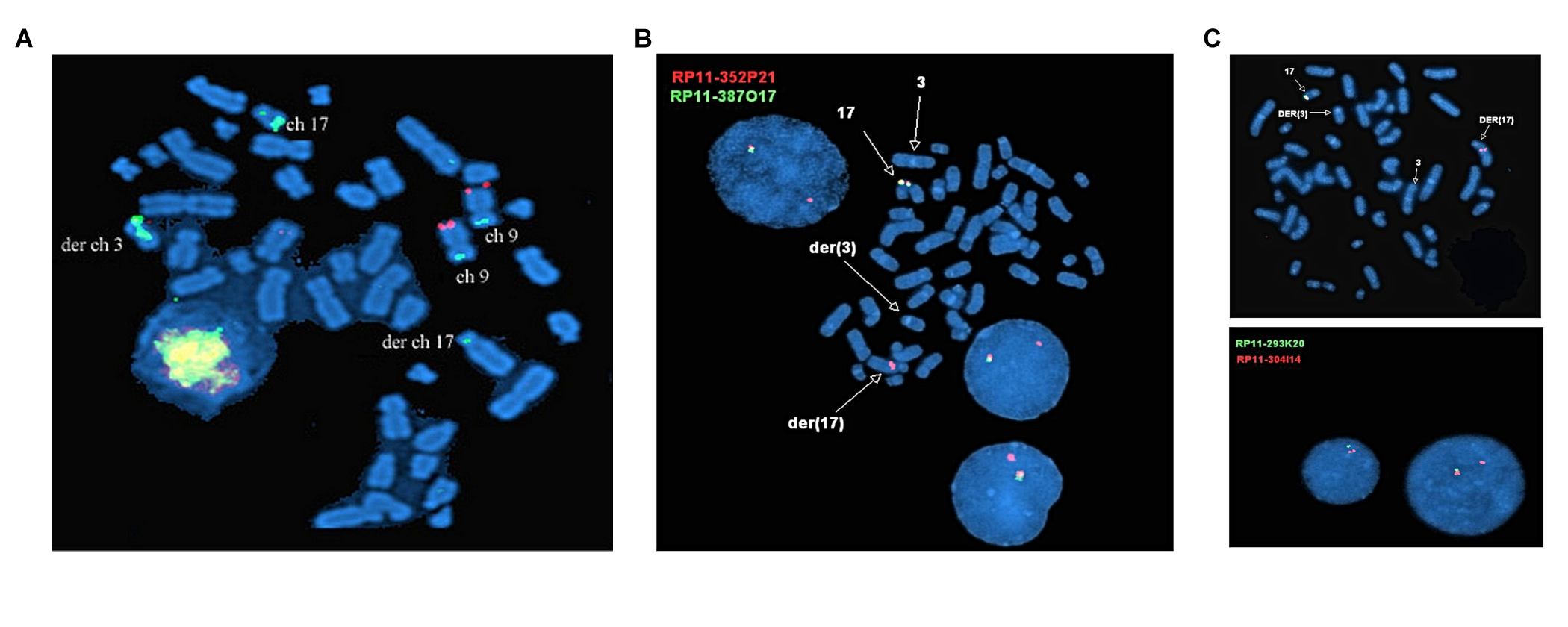

Supplement: Figure S7 — Telomere FISH and FISH using BAC clones spanning chromosome 17q24.2-24.3 to detect the 1.3 Mb cryptic deletion in sporadic CGHT. (A) Telomere FISH was performed to test possible deletions at the end of chromosome 17q using a commercially available probe mix for 17q (green), 17 centromere (green), 9p (green), and 9q (red). Note the presence of the green signal on the derived chromosome 3 indicating that the telomere of chromosome 17 was not deleted in the t3;17 rearrangement. (B–C) FISH using BAC clones on chromosome 17q24.2-q24.3 revealed a cryptic deletion at the breakpoint of chromosome 17. Metaphase spreads and interphase nuclei show only one signal for clones RP11-387O17 (green) (B) and RP11-293K20 (green) (C), which hybridize to the deleted 1.3 Mb portion of chromosome 17q24.2-24.3. (TIF) [file pgen.1004333.s007.tif]

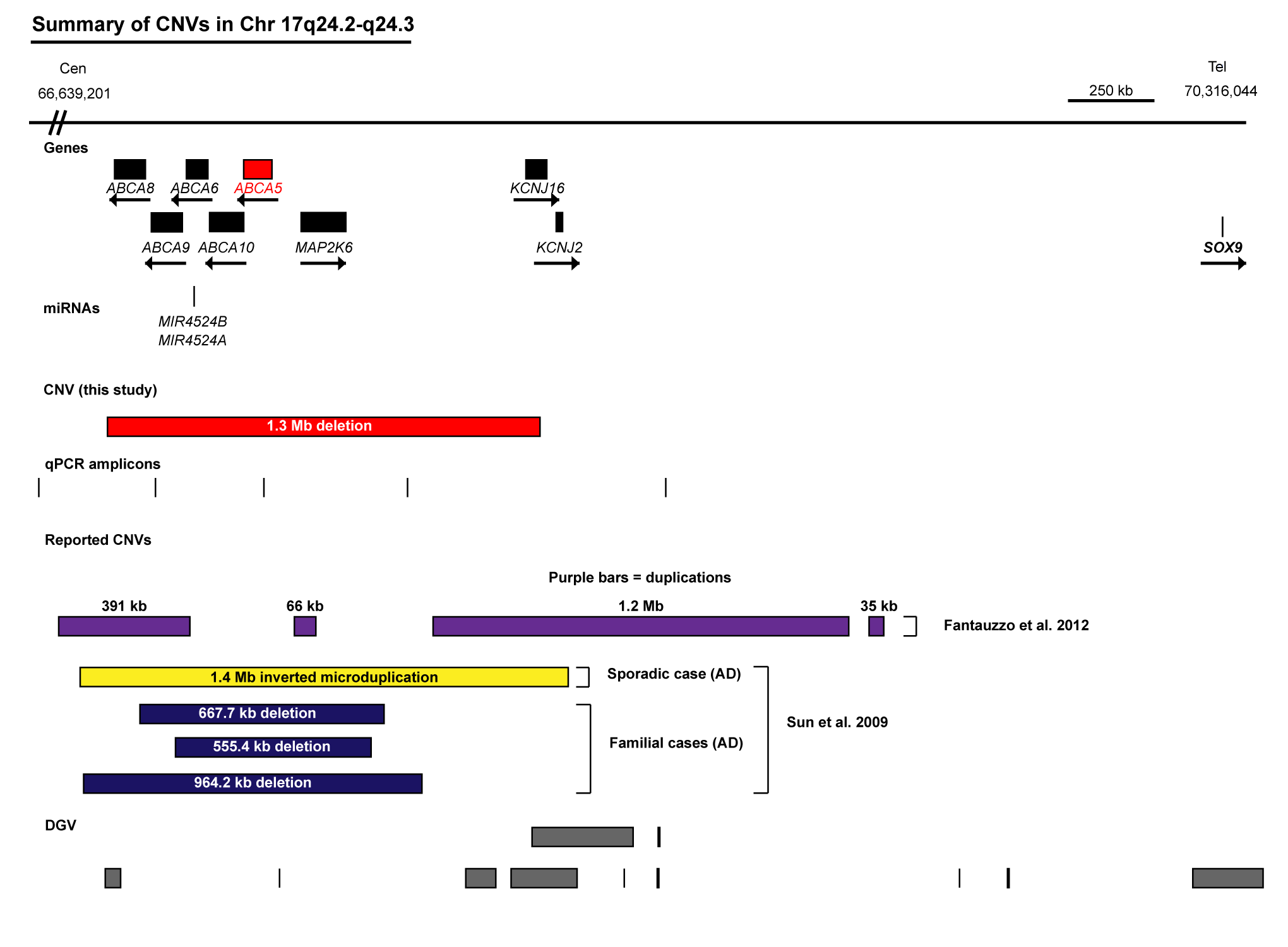

Supplement: Figure S8 — Summary of CNVs within the chr17q24.2-24.3 region identified in autosomal dominant and sporadic cases of CGHT illustrates that ABCA5 is located in the minimal common region. ABCA5 (red box) and the other genes in the surrounding region (black boxes) as well as direction of transcription (arrows) are indicated. Nature of the variants (duplications, deletions) previously reported as well as identified in this study is indicated as well as the sizes and corresponding references. qPCR amplicons are represented by vertical lines, where two amplicons flank the 1.3 Mb deleted region (red box) and three amplicons lie within it. Database of Genomic Variants (DGV) alterations are indicated as gray boxes. All variants lie 1–2 Mb upstream of the SOX9 gene. All coordinates reference the UCSC Genome Browser human reference genome hg19. (TIF) [file pgen.1004333.s008.tif]
